# THetA: inferring intra-tumor heterogeneity from high-throughput DNA sequencing data

**DOI:** 10.1186/gb-2013-14-7-r80

**Published:** 2013-07-29

**Authors:** Layla Oesper, Ahmad Mahmoody, Benjamin J Raphael

**Affiliations:** 1 Department of Computer Science, Brown University, 115 Waterman Street, Providence, RI 02912, USA; 2 Center for Computational Molecular Biology, Brown University, Box 1910, Providence, RI 02912, USA

**Keywords:** Cancer genomics, intra-tumor heterogeneity, DNA sequencing, tumor evolution, algorithms

## Abstract

Tumor samples are typically heterogeneous, containing admixture by normal, non-cancerous cells and one or more subpopulations of cancerous cells. Whole-genome sequencing of a tumor sample yields reads from this mixture, but does not directly reveal the cell of origin for each read. We introduce THetA (Tumor Heterogeneity Analysis), an algorithm that infers the most likely collection of genomes and their proportions in a sample, for the case where copy number aberrations distinguish subpopulations. THetA successfully estimates normal admixture and recovers clonal and subclonal copy number aberrations in real and simulated sequencing data. THetA is available at http://compbio.cs.brown.edu/software/.

## Background

Cancer is a disease driven in part by somatic mutations, which accumulate during the lifetime of an individual. The clonal theory of cancer progression [1] states that the cancerous cells in a tumor are descended from a single founder cell and that descendants of this founder cell acquired multiple mutations beneficial for tumor growth through multiple rounds of selection and clonal expansion. A tumor is thus a heterogeneous population of cells, each cell potentially containing a different complement of somatic mutations. These include both clonal mutations from the founder cell or early rounds of clonal expansion and subclonal mutations that occurred after the most recent clonal expansion. Alternatively, subclonal mutations may suggest that the tumor is polyclonal, consisting of subpopulations of cells that are not all descended from a single founder cell [2].

High-throughput DNA sequencing technologies are now giving an unprecedented view of this intra-tumor mutational heterogeneity [3]. However, nearly all recent cancer sequencing projects generate DNA sequence from tumor samples consisting of many cells - including both normal (non-cancerous) cells and one or more distinct populations of tumor cells. The tumor purity of a sample is the fraction of cells in the sample that are cancerous, and not normal cells. If a sample has a low tumor purity, then the power to detect all types of somatic aberrations in the cancer genomes is reduced. For example, lower tumor purity attenuates copy number ratios or allele frequencies away from the values expected with integral copy numbers. Methods to detect somatic copy number aberrations or loss of heterozygosity (LOH) from SNP array data or array comparative genomic hybridization (aCGH) data must account for this issue [4-9]. In addition, many algorithms for identifying somatic single-nucleotide mutations from DNA sequence reads implicitly or explicitly rely on an estimate of tumor purity. For example, the VarScan 2 program [10] uses an estimate of tumor purity as input to calibrate the expected number of reads that contain a somatic mutation at a locus.

Traditionally, tumor purity was assessed by visual analysis of tumor cells, either manually by a pathologist or via image analysis [11]. Recently, methods such as ASCAT [12] and ABSOLUTE [13] were introduced to estimate tumor purity directly from SNP array data. Both of these methods utilize the presence of copy number aberrations in cancer genomes to estimate both tumor purity and tumor ploidy, which is the number of copies of segments of chromosomes or entire chromosomes. Tumor purity and tumor ploidy are intertwined; for example, a heterozygous deletion of one copy of a chromosome in a 100% pure tumor sample (containing one cancer genome) could also be explained as a homozygous deletion in a 50% pure tumor sample (containing one cancer genome). Thus, it is necessary to estimate tumor purity and ploidy simultaneously, but this is a subtle and difficult problem. ASCAT and ABSOLUTE address this problem by estimating the average ploidy over the entire cancer genome. These estimates of tumor purity and average ploidy are then used in a second step to derive copy number aberrations.

Both ASCAT and ABSOLUTE have been shown to yield accurate estimates of tumor purity, achieving in some cases better estimates than via pathology or other techniques. However, these methods also have important limitations. First, the mathematical models used by ASCAT and ABSOLUTE are optimized for SNP array data, as we detail below. While these methods may be adapted to run on DNA sequencing data (for example, for ABSOLUTE see [14] and for ASCAT see below), the underlying mathematical model used by both methods does not adequately describe the characteristics of sequencing data. Second, both of these methods apply various heuristics in their estimation procedures, such as rounding copy numbers to the closest integer [12] and do not directly infer integer copy numbers for each segment of the genome during the estimation. Finally, both methods do not explicitly identify multiple tumor subpopulations, and instead infer only a single tumor subpopulation. For example, ABSOLUTE [13] classifies copy number aberrations as outliers if they are not clonal, but does not refine these outliers into subpopulations. If a tumor sample consists of multiple tumor subpopulations, then considering only a single tumor population may yield inaccurate estimates of tumor purity, as we show below.

High-throughput DNA sequencing data is much higher resolution data than SNP arrays, and provides the opportunity to derive highly accurate estimates of both tumor purity and the composition of tumor subpopulations. For example, the number of reads containing a somatic single-nucleotide mutation at a locus provides - in principle - an estimate of the fraction of cells in a tumor sample containing this mutation. However, three interrelated factors complicate this analysis: (1) The number of reads supporting a somatic single-nucleotide mutation has high variance, implying that an estimate of the allele frequency will be highly unreliable at the modest coverages (30× to 40×) employed in nearly all current cancer sequencing projects. (2) Somatic mutations may be present in only a fraction of tumor cells. (3) Somatic copy number aberrations (nearly ubiquitous in solid tumors) alter the number of copies of the locus containing the mutation. While the first issue might be addressed in part by clustering allele frequency estimates across the genome [15-17], the second and third issues complicate such a clustering. Recent methods for analyzing tumor composition from DNA sequencing data either ignore copy number aberrations [17] or use iterative approaches [18] or other approximations [12,13], and do not formally model the generation of DNA sequencing data from a mixture of integral copy numbers for each genomic segment.

Beyond the estimation of tumor purity and ploidy, it is desirable to identify subclonal aberrations, which can provide information on the age or history of the tumor [19], and can yield further insight into tumors that fail to respond to treatment or metastasize [19-21]. However, even with a pure tumor sample, characterizing subclonal mutations is a challenge. Tolliver *et al. *[22] infer subclonal copy number aberrations by comparing aberrations across different individuals, thus assuming that the progression of somatic copy number aberrations is conserved across individuals. Gerlinger *et al. *[23] recently demonstrated the extent of subclonal mutations by sequencing multiple (spatially separated) samples from a tumor, complementing earlier studies of heterogeneity using microarray-based techniques [24]. In another approach, Ding *et al. *[17] used a targeted ultra-deep sequencing (1,000 × coverage) approach to estimate allele frequencies for relapse mutations in acute myeloid leukemia (AML). In another recent study, Nik-Zainal *et al. *[25] used a SNP array based estimate of tumor purity [12] followed by extensive manual analysis of somatic mutations to identify a clonal (majority) population and a number of subclonal populations in each of several breast cancer genomes. Ultimately, single-cell sequencing techniques promise to provide a comprehensive view of cancer heterogeneity [26-29], but these techniques presently require specialized DNA amplification steps, which can introduce artifacts and also incur higher costs because they sequence many cells. Thus, the problem of the simultaneous estimation of and correction for tumor purity as well as the identification of clonal and subclonal mutations will remain a challenge for the majority of cancer sequencing projects.

In this paper, we introduce Tumor Heterogeneity Analysis (THetA), an algorithm that infers the most likely collection of genomes and their proportions from high-throughput DNA sequencing data, in the case where copy number aberrations distinguish subpopulations. In contrast to existing methods, we formulate and optimize an explicit probabilistic model for the generation of the observed tumor sequencing data from a mixture of a normal genome and one or more cancer genomes, each genome containing integral copy numbers of its segments. Specifically, we derive and solve the maximum likelihood mixture decomposition problem (MLMDP) of finding a collection of genomes - each differing from the normal genome by copy number aberrations - whose mixture best explains the observed sequencing data. Thus, we generalize the problem of estimating tumor purity to the problem of determining the proportions of normal cells and any number of tumor subpopulations in the sample.

Our formulation and solution of the MLMDP leverages the fact that copy number aberrations create a strong signal in DNA sequencing data: even relatively small copy number aberrations cause deviations in the alignments of thousands to millions of reads. Thus, in contrast to single-nucleotide mutations, where there is high variance in the number of reads at each position, many measurements (reads) are perturbed for each copy number aberration. Thus, each copy number aberration provides many data points for deconvolution of the tumor genome mixture. We show how to solve the MLMDP as a collection of convex optimization problems. THetA is the first algorithm - to our knowledge - that automatically identifies subclonal copy number aberrations in whole-genome sequencing data from mixtures of more than two genomes. Moreover, in the case of an admixture between a single (clonal) cancer population and normal cells, THetA runs in polynomial time; it is the first rigorous and efficient algorithm for simultaneously estimating tumor purity and inferring integral copy numbers.

We apply our THetA algorithm to simulated data and to real DNA sequencing data from breast tumors sequenced at approximately 188× and approximately 40× coverage from [25]. We quantify the normal cell admixture in each tumor, outperforming other algorithms for this task. We also demonstrate that allowing only one tumor subpopulation may lead to highly inaccurate tumor purity estimates, and subsequent failure to detect clonal and subclonal copy number aberrations. In the 188× sequenced tumor, we identify both clonal and subclonal tumor cell populations, each containing unique copy number aberrations. Our results recapitulate most of the findings reported in [25] for this sample, but also have some distinct differences, which are supported by the sequencing data. In one of the 40× sequenced tumors, we identified two previously unreported tumor subpopulations, demonstrating the ability to identify intra-tumor heterogeneity, in particular subclonal aberrations, at the modest sequence coverages that are the current standard in cancer sequencing studies.

## Results

### Maximum likelihood mixture decomposition problem

First, we will formulate the maximum likelihood mixture decomposition problem of finding the most likely mixture of tumor cell populations from a sequenced tumor sample. We assume that sequenced reads from a tumor sample are aligned to the reference human genome, the first step in cancer genome sequencing analysis [30,31]. Typically, a matched normal genome is also sequenced to distinguish somatic mutations from germline variants. We focus on copy number aberrations in order to estimate tumor purity and subpopulations. Thus, we assume that a cancer genome differs from the reference genome by gains and losses of segments, or intervals, of the reference genome. These intervals are identified by examining the density, or depth, of reads aligning to each location in the reference [32-34], and/or by clustering discordant paired reads that identify the breakpoints of copy number aberrations or other rearrangements [35-40]. Following this analysis, the reference genome is partitioned into a sequence **I **= (*I*_1_, ..., *I_m_*) of non-overlapping intervals. We represent a cancer genome by an interval count vector **c **= (*c*_1_, ..., *c_m_*), where *c_j _*∈ ℕ is the integer number of copies of interval *I_j _*in the cancer genome. From the sequencing of a tumor sample, we observe a read depth vector **r **= (*r*_1_, ..., *r_m_*), where *r_j _*∈ ℕ is the number of reads with a (unique) alignment within *I_j_*.

**Figure 1:**
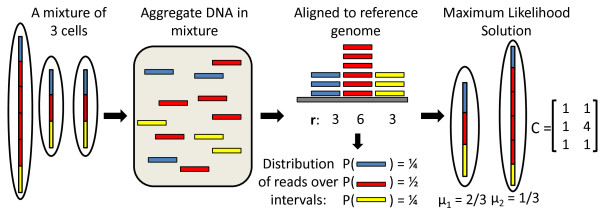
**Algorithm overview**. A mixture of three subpopulations with two distinct genomes: a normal genome (represented here with one copy of each interval for simplicity), and an aneuploid genome with a duplication of one interval (red). If reads are distributed uniformly over the aggregate DNA in the sample, then the observed distribution of reads over the blue, red and yellow intervals will follow a multinomial distribution with parameter $\hat{C\mu }$. Here **C **is the interval count matrix giving the integral number of copies of each interval in each genome in the mixture, and *μ *is the genome mixing vector giving the proportion of each subpopulation in the mixture. We find the pair (**C**, *μ*) that maximizes the likelihood of the observed read depth vector **r**.

A tumor sample is a mixture of cells that contain different collections of somatic mutations, and in particular somatic copy number aberrations. We assume that the tumor sample is a mixture of *n *subpopulations, including a subpopulation of normal cells and one or more subpopulations of cancer cells. Each subpopulation has a distinct interval count vector representing the genome of the subpopulation. Thus, we represent a tumor sample  by: (1) an *m *× *n *interval count matrix **C **= [*c_jh_*], where *c_jh _*∈ ℕ is the number of copies of interval *I_j _*in the *h*th distinct subpopulation; and (2) a genome mixing vector *μ *∈ ℝ*^n ^*where *μ_h _*is the fraction of cells in  from the *h*th subpopulation. Given a read depth vector **r **derived from the sequence of , our goal is to identify the underlying interval count matrix **C **and genome mixing vector *μ *that best describe **r **(Figure 1). We formulate the following problem.

**Maximum likelihood mixture decomposition problem (MLMDP)**. *Given an interval partition ***I ***of a reference genome and an associated read depth vector ***r ***derived from a tumor sample , find the underlying interval count matrix ***C ***and genome mixing vector μ that maximize the likelihood P*(**r***|***C***, μ*).

In the Materials and methods section below, we derive the probability *P*(**r**|**C**, *μ*) in the MLMDP. In brief, under the usual assumptions for DNA sequencing, the probability *p_j _*that a read that aligns to an interval *I_j _*is equal to the fraction of the total DNA in the sample originating from interval *I_j_*. Hence, the probability *P*(**r**|**C**, *μ*) of the observed read depth vector **r **follows a multinomial distribution determined by the interval count matrix **C **and genome mixing vector *μ*. We emphasize that the multinomial distribution models the fact that the number of reads aligning to each interval are not independent random variables, but rather are dependent on the number of copies (ploidy) of each interval in the cancer genome(s) (please see Additional file 1, Section A). In contrast to our probabilistic model for DNA sequencing data, other methods for estimating tumor purity and ploidy [12,13] do not model the data as an observation from an experiment. Rather, they assume that the observed copy number ratio of an interval (or probe) is the ratio of the expected value of the tumor copy number and the expected value of the normal copy number (please see Additional file 1, Section B). Thus, they implicitly assume that the observed data is an average over many experiments.

### Solving the maximum likelihood mixture decomposition problem

We now give an overview of our algorithm for solving the instance of the MLMDP where *P*(**r**|**C**, *μ*) is the multinomial probability described above. Further details are in the Materials and methods section.

#### Restricting the space of interval count matrices

In practice, the interval count matrix **C **is not allowed to be any integer-valued matrix. There are three natural constraints on the interval count matrix: (1) One component of the tumor sample is the normal genome. Thus, we set the first column **c**_1 _= (2, 2, ..., 2)^T^, the vector whose entries are all two. (2) The number *n *of subpopulations is less than the number *m *of intervals. (3) The copy numbers of the intervals are integers between 0 and *k*, inclusive, where *k *≥ 2. We let $C_{m,n,k}$ denote the set of all matrices satisfying these properties.

#### A convex optimization algorithm

We wish to find the interval count matrix $\text{C}\in C_{m,n,k}$ and the genome mixing vector *μ *that maximize the multinomial likelihood *P*(**r**|**C**, *μ*). However, this optimization problem is not straightforward to solve because it contains both integer-valued variables (entries of **C**) and real-valued variables (entries of *μ*). We show that a special coordinate transformation allows the MLMDP to be solved as a disjunction of constrained convex optimization problems by enumerating the possible interval count matrices and solving a separate convex optimization problem for each such **C **(see Materials and methods). Since the number of possible matrices **C **grows exponentially with *m *and *n*, this brute-force strategy approach will not scale well beyond small values of *n *subpopulations and *m *intervals. In a special, but important, case where a sample contains a single clonal tumor population along with a normal admixture (that is, *n *= 2), we show how to further restrict the space of possible interval count matrices **C**, and obtain an efficient algorithm (polynomial time in *m*) for the MLMDP. The runtime for our algorithm depends on the number of intervals *m *and maximum copy number *k *in the input. Simulations with *m *= 39 and *k *= 3 (described below) run in 1 to 2 minutes on a standard desktop, while increasing to *k *= 5 increases the runtime to approximately 25 to 40 minutes.

#### Selecting a solution

Two additional issues to be addressed in deriving a solution are: (1) how to select from multiple optimal solutions and (2) how to choose the number *n *of tumor subpopulations in the mixture. We note that tumor sequencing data alone does not distinguish between different optimal solutions with the same maximum likelihood. In mathematical terms, this is because only the parameter of the multinomial distribution is identifiable from the observed read depth vector **r**. Thus, we cannot distinguish between pairs (**C**, *μ*) and (**C**', *μ*') of interval count matrices and genome mixing vectors that give the same multinomial parameter. Our algorithm THetA has options to return the complete family of optimal solutions, or to limit to solutions with a baseline copy number of the clonal tumor population (see Materials and methods).

Regarding the second issue, note that the likelihood *P*(**r**|**C**, *μ*) increases as the number *n *of tumor subpopulations in the mixture increases: indeed the observed read depth vector can be fitted 'perfectly' by placing each copy number aberration in its own tumor subpopulation. However, mixtures with larger *n *also have greater model complexity (that is, more parameters). We use a model selection criterion based on the Bayesian information criterion (BIC) to select a model with a balance between higher likelihood and lower model complexity in order to avoid overfitting.

### Evaluation on simulated cancer genomes

#### Normal admixture: single cancer genome

Using two different sets of simulated data, we compared our THetA algorithm to three other methods for estimating tumor purity and ploidy: ASCAT [12], ABSOLUTE [13] and CNAnorm [18]. ASCAT and ABSOLUTE jointly estimate tumor purity and ploidy, and were originally designed for SNP array data. While both can be adapted to run on DNA sequencing data, they do not formally model this type of data, as noted above. CNAnorm is designed for DNA sequencing data, but rather than allowing tumor purity and tumor ploidy to inform each other, it uses an iterative approach that separately infers purity and copy numbers. In some instances, CNAnorm relies on the user manually entering the most abundant ploidy.

As noted above, there are multiple optimal solutions with the same maximum likelihood. CNAnorm [18] and ASCAT [12] use *ad hoc *criteria to return only a single purity estimate, and ABSOLUTE [13] uses external cancer karyotypes to select from multiple possible solutions. To compare THetA to these other methods, we must select a single pair (**C**, *μ*) from the set returned by THetA as a representative sample reconstruction. For all simulations, we chose the pair (**C**, *μ*) that maximizes the total length of all genomic intervals in the tumor genome with copy number 2, the expected copy number of the normal genome for humans. We note that this decision applies only to these simulations - for real sequencing data the set of all equally like solutions is returned by THetA from which a user may select one using additional information about the sample under consideration. For further details about the other algorithms please see Additional file 1, Sections K and L.

For our first set of simulations, we generated a cancer genome consisting of chromosome arm copy number aberrations. The copy number for each non-acrocentric chromosome arm was chosen uniformly at random from the range 0 (that is, homozygous deletion) through *k *> 2 (amplification), up to a specified maximum copy number *k*. While real cancer genomes may have copy numbers larger than the maximum value (*k *= 7) considered in these simulations, such high amplitude amplifications are generally focal events. We emphasize that it is not necessary to use all copy number aberrations to infer the tumor composition; for example, if there are a sufficient number of arm-level copy number aberrations, these may suffice. We then created a random mixture of this cancer genome and a 'matched normal' genome and simulated a read depth vector **r **for the mixture, adding noise according to the read depth estimation error *φ*. The parameter *φ *models errors in the sequencing and analysis process, and we estimated from real sequencing data that *φ *is in the range from 0.01 to 0.04 (please see Additional file 1, Section J and Figure S3). Since the ASCAT algorithm uses SNP array data, we also simulated SNP array data from our mixture. Further details of the simulations are in Additional file 1, Section I.

Table 1 shows how the four algorithms performed on the simulated datasets with interval count matrix $\text{C}\in C_{39,2,k}$ and mixing vector *μ *and read depth estimation error *φ *= 0.03. For each value of *k*, the maximum copy number, 20 simulated datasets were generated. The percentage correct **C **is the percentage of datasets where the inferred interval count matrix C* exactly equals the true simulated matrix **C **for the sample. The copy number error is $\frac{1}{m\left(n-1\right)}{\left|C-C^{*}\right|}_{2}$, that is the average error per copy number estimate made, or per entry in **C**, where error is the Euclidean distance between **C **and C*. The purity error is $\left|\mu_{2}-\mu_{2}^{*}\right|$, that is the distance between the true and inferred tumor purity. We only calculated results for CNAnorm where the inferred purity was <100% (there were between 12 and 15 trials for each *k*). Additional file 1, Figure S4 illustrates the results obtained by each algorithm on one of the datasets when *k *= 7.

**Table 1:** Performance of the algorithms on simulated data with one tumor population (*n *= 2)

	% correct C				Copy number error (median)				Purity error (median)			
*k*	THetA	ASCAT	CNAnorm	ABSOLUTE	THetA	ASCAT	CNAnorm	ABSOLUTE	THetA	ASCAT	CNAnorm	ABSOLUTE
3	100.0	85.0	40.0	70.0	0.0	0.0	0.103	0.000	0.004	0.040	0.068	0.010
4	90.0	55.0	8.3	50.0	0.0	0.0	0.163	0.013	0.004	0.037	0.064	0.010
5	85.0	50.0	6.7	15.0	0.0	0.013	0.185	0.160	0.004	0.062	0.038	0.075
6	55.0	40.0	0.0	15.0	0.0	0.026	0.291	0.433	0.006	0.063	0.066	0.157
7	30.0	15.0	0.0	10.0	0.031	0.036	0.445	0.471	0.005	0.069	0.108	0.149

For this first set of simulations, we found that our THetA algorithm computes both **C **and *μ *very accurately over a range of copy numbers *k*. In particular, THetA outperforms CNAnorm, ABSOLUTE and ASCAT despite the fact that ASCAT uses additional information (allele frequencies) that THetA does not consider. Even with high amplitude copy number aberrations (*k *= 7) THetA on average estimates tumor purity within 0.5% of the true purity, compared to 6.9%, 10.8% and 14.9% by ASCAT, CNAnorm and ABSOLUTE respectively. Even when THetA does not estimate all copy numbers across the genome correctly, it estimates most copy numbers correctly and estimates the copy number correctly for more segments than the other algorithms (see Additional file 1, Figure S4). Further results comparing THetA to CNAnorm for different read depth estimation errors are in Additional file 1, Figure S5.

**Figure 2:**
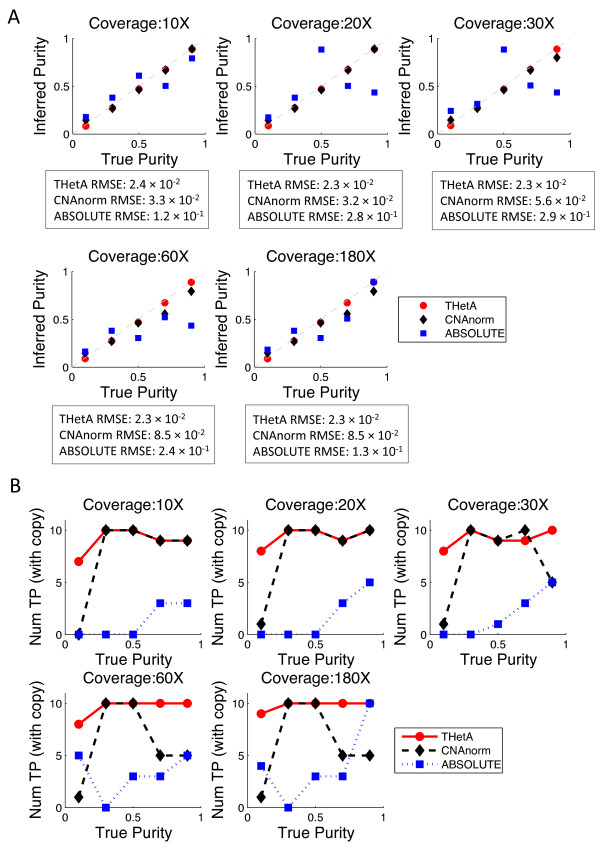
**Comparison of THetA to CNAnorm and ABSOLUTE on simulated mixtures from real sequencing data**. **(A) **Comparison of true and inferred tumor purity by THetA, CNAnorm and ABSOLUTE on simulated mixtures of DNA sequencing data from an acute myeloid leukemia sample and a matched normal sample. Gray dashed line indicates True Purity = Inferred Purity. Below each plot are the root mean squared errors (RMSEs) for each method. **(B) **Comparison of the number of copy number aberrations correctly predicted (defined as 50% reciprocal overlap in position and correct integral copy count) by each method for varying tumor purity and sequence coverage. Num TP is the number of true positive copy number aberrations predicted. In most cases, THetA outperforms both CNAnorm and ABSOLUTE. Similar results counting aberrations with correct position (with 50% reciprocal overlap) but allowing for difference between true and predicted copy number are in Additional file 1, Figure S7. RMSE: root mean squared error.

We also compared THetA, CNAnorm, and ABSOLUTE using a second set of simulated mixtures of tumor and normal cells created using real sequencing data from an AML tumor sample and matched normal sample (TCGA-AB-2965) from The Cancer Genome Atlas (TCGA) [41]. This sample was chosen due to its high purity (approximately 95% pure) and lack of copy number aberrations. We spiked 10 copy number variants of length 2.5 Mb at random non-overlapping positions in Chr20 (excluding the centromere) into the tumor genome. As in the first set of simulations, the copy number for each variant was chosen uniformly at random from the range 0 (that is, homozygous deletion) through *k *> 2 (amplification), up to a specified maximum copy number *k *= 5. (We did not run ASCAT on this simulated data since this algorithm was designed only for microarray data.) We again found that THetA outperforms both CNAnorm and ABSOLUTE on all measures (Figure 2). In particular, THetA estimates the sample purity with an order of magnitude better accuracy (using the root mean squared error as a metric of comparison as was done for ABSOLUTE [13]), and consistently identifies more true copy number aberrations than the other algorithms across different purity values and sequencing coverage. In particular, THetA identifies 7.4 and 2.2 more copy number aberrations, on average, than ABSOLUTE and CNAnorm, respectively, across all purity values at 30× sequencing coverage. Even when we relax the requirement that a copy number aberration must be predicted with the correct copy number, and instead count any non-normal copy number as correct, THetA still outperforms the other algorithms (see Additional file 1, Figure S7). Further details of the simulations are in Additional file 1, Section I.

#### Mixture of tumor subpopulations

We next evaluated the performance of THetA on a simulated mixture containing two subpopulations of tumor cells with different copy number aberrations and an admixture with normal cells. Thus, there were three distinct subpopulations in the mixture (*n *= 3). Our method for constructing the simulated data was the same as for the first set of simulations, as described in the previous section, with a fixed read depth estimation error of *φ *= 0.02 along with a few minor changes (see Additional file 1, Section I).

Table 2 shows how the three algorithms performed on the simulated datasets with interval count matrix **C **∈ $C_{m,3,3}$ and mixing vector *μ *and read depth estimation error *φ *= 0.02. The percentage correct **C **and copy number error are defined as for Table 1. We defined the purity error as the distance between the true and predicted fraction of tumor cells in the sample. Thus, purity error is ${\left|\left(1-\mu_{1}\right)-\left(1-\mu_{1}^{*}\right)\right|}_{2}$, as the proportion of tumor cells in the sample is 1- *μ*_1_. Since CNAnorm and ABSOLUTE are not able to infer multiple subpopulations, their percentage correct **C **= 0, and we list only their purity estimates. We only calculated results for CNAnorm where the inferred purity was <100% (there were between 14 and 18 trials for each *m*).

**Table 2:** Performance of the algorithms on simulated data with two tumor populations (*n *= 3)

	% correct C	Copy number error (median)	Purity error (median)		
*m*	THetA	THetA	THetA	CNAnorm	ABSOLUTE
6	35.0	0.118	0.081	0.202	0.458
8	45.0	0.075	0.052	0.276	0.477
10	35.0	0.071	0.055	0.177	0.434
12	45.0	0.059	0.036	0.301	0.547

While the performance of THetA was less precise in estimating all copy numbers (that is, the entries in **C**) exactly than for the tumor with a normal admixture (*n *= 2), THetA maintains a good level of accuracy as the estimates are near the true interval copy numbers. THetA correctly computes on average 94% of the copy numbers across all subpopulations in the mixture when there are *m *= 12 intervals with varying copy number in the subpopulations. THetA also estimates the tumor purity with good accuracy (within 3.6% of the true purity when *m *= 12), whereas both CNAnorm and ABSOLUTE gravely misestimate the tumor purity by 30.1% and 54.7%, respectively. One possible explanation for these errors is that both of these methods do not account for multiple subpopulations in the sample and therefore tend to report tumor purity as either the fraction of the sample representing the largest subpopulation, or as an average of the fractions of all tumor subpopulations. Thus, THetA successfully recovers a complex mixture of two tumor subpopulations and a normal cell admixture directly from the observed read depth.

### Results from breast cancer sequencing data

We analyzed Illumina paired-end sequencing data from three breast cancer genomes and their matched normal samples from [25]. We downloaded the data from the European Genome-phenome Archive (accession number EGAD00001000138). This includes two samples that were sequenced to a depth of approximately 40× coverage and one sample, PD4120a, that was sequenced with approximately 188× coverage. We used the BIC-Seq segmentation algorithm [32] to partition the 22 autosomes into intervals according to read depth. We formed an interval count vector from all intervals longer than 50 kb, focusing on these longer genomic intervals because their observed read depth will have lower variance (see Additional file 1, Figure S11). Most intervals removed in this step are relatively short for the samples analyzed. For the two 40× coverage genomes changing this cutoff to 10 kb resulted in the same partition as when 50 kb was used. For the 188× genome we only removed nine intervals from consideration when the threshold was 50 kb and seven when the threshold was 10 kb. The results from THetA are identical for the two different sets of intervals. We assume that most of the tumor genome does not undergo copy number aberrations, and thus the mode of the read depth vector is a normal baseline. We set lower and upper bounds on the copy number for each interval from this baseline. For further details, see Additional file 1, Section N.

#### Breast tumor: 188× sequence coverage

We analyzed the 188× sequenced tumor PD4120a using our THetA algorithm. We consider that the mixture contains a normal admixture with a single tumor subpopulation (*n *= 2) and a normal admixture with two distinct tumor subpopulations (*n *= 3). This sample was extensively annotated by [25] and thus acts a positive control for THetA.

Table 3 shows the tumor purity and copy number aberrations identified by the various algorithms on the 188× coverage breast cancer genome (sample PD4120a). Assuming a single tumor subpopulation admixed with normal cells (*n *= 2), THetA's estimate of tumor purity (65.7%) and inferred copy number aberrations are very close to those obtained by CNAnorm [18] (67.2%), ASCAT (66.0%) [12] and ABSOLUTE (65%) [13]. However, all of these estimates are lower than the tumor purity of 70% reported by [25], who identified a second tumor subpopulation in the sample (see below). Because ABSOLUTE, ASCAT, CNAnorm and THetA (with *n *= 2) do not model multiple tumor subpopulations, their reported tumor purities are an average of the fraction of aberrant cells amongst the different subpopulations in the tumor sample, and thus generally smaller than the tumor purity estimate obtained when we allow more than one tumor subpopulation (see below). In addition, we note that ASCAT used additional information (B-allele frequencies), while THetA, CNAnorm and ABSOLUTE used only read depth. The identified aberrations do not distinguish between those in different subpopulations, but do include several previously reported in breast cancer [42-47]. We also ran THetA using chromosome arms as the intervals, rather than the BIC-Seq intervals. Using chromosome arms, we estimated a similar sample purity of 61.7% and predicted the same set of copy number aberrations as with the BIC-Seq intervals.

**Table 3:** Comparison of various algorithms on the 188× coverage breast cancer genome

Sample PD4120a			
**Algorithm**	**% normal admixture**	**Clonal (% tumor purity)^a^**	**Subclonal (%)^a^**

THetA, *n *= 2	34.3%	Del: 1p, 4q, 13, 16q, 22q	-
(segmentation)		+1: 1q	
		(65.7%)	

THetA, *n *= 2 (chromosome arms)	38.3%	Del: 1p, 4q, 13, 16q, 22q	-
		+1: 1q	
		(61.7%)	

CNAnorm^b^ (chromosome arms)	32.8%	Del: 1p, 4q, 13, 16q, 22q	-
		+1: 1q	
		(67.2%)	

ASCAT^c^	34%	Del: 1p, 4q, 13, 16q, 22q	-
(virtual SNP array)		+1: 1q, 17q, 18, 19, 20	
		(66.0%)	

ABSOLUTE^d ^(segmentation)	35%	(65.0%)	-

THetA	28.0%	Del: **1p**, 4q, **16q**, **22q12.2-**	Del: 13, 22q11.2-12.1
*n *= 3		**13.3**	+1: **1q**
		(72.0%)	(61.9%)
			Del: 8, 11, 12, 14, 15
			(10.1%)
			*Del: 2, 7, **4p**, 6*, *9, 18, 21*

Nik-Zainal *et al*. (2012)	30%	Del: 4q	Del: 13, **t(1;22)**
[25]		+1: **1q**	(47.6%)
		(70.0%)	**Tetraploid **with:
			Del(-2): 2, 7
			Del(-1): 6, 8, 9, 11, 12,
			14, 15, 18, 21
			(9.8%)

a Entries in bold are differences between THetA and [25]. Entries in italics were not input to the *n *= 3 THetA analysis but were inferred using THetA's output.

^b ^When CNAnorm was run using BIC-Seq intervals the normal admixture was estimated at 6.7%, therefore we report results from CNAnorm using chromosome arms. CNAnorm does not return integer copy numbers - and thus we report aberrations where the returned copy number was within 0.15 of the nearest integer, other aberrations were considered inconclusive.

^c ^For ASCAT we use virtual SNP array data as input. ASCAT performs its own segmentation; we list only the large aberrations.

^d ^We report here the maximum likelihood solution returned by ABSOLUTE when considering only karyotypes. When considering only somatic copy number aberrations or a combination, ABSOLUTE infers a tetraploid solution. For this sample, ABSOLUTE returns copy numbers for only a subset of the input intervals, so we do not report specific copy number aberrations predicted.

**Figure 3:**
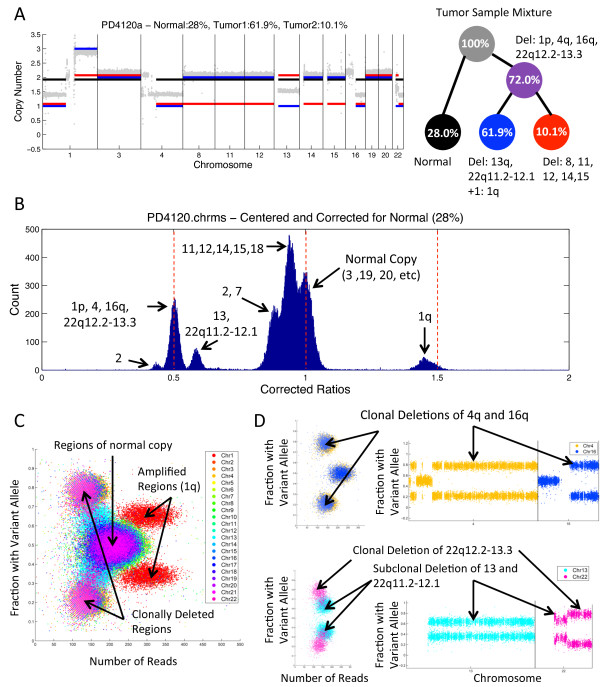
**Analysis of the 188× coverage breast tumor PD4120a**. **(A) **(Left) Read depth ratios (gray) and the copy number aberrations inferred by our algorithm when *n *= 3 including the normal population (black), dominant (clonal) tumor population (blue) and subclonal tumor population (red). (Right) A reconstruction of the tumor mixture with the inferred aberrations and estimated fraction of cells in each subpopulation. **(B) **Read depth ratios in 50 kb intervals after centering so chromosome 3 has a mean of 1 and correcting for 28% normal admixture using a simple linear scaling. **(C) **Virtual SNP array results showing distinct clusters of regions according to the number of reads containing each SNP and fraction of reads supporting the variant allele. **(D) **Virtual SNP array data comparing variant allele fractions and read counts for chromosomes 4 and 16. This data demonstrate that both chromosomes have undergone the same type of copy number aberration, which we predicted to be a clonal deletion in 72% of cells in the sample. **(E) **Virtual SNP array data for chromosomes 13 and 22. Chromosome 22q11.2-12.1 and chromosome 13 appear to be affected by the same type of aberration, which we predicted to be a subclonal deletion in 61.9% of cells in the sample. In contrast, 22q12.2-13.3 is different, and the data are consistent with a clonal deletion. See Additional file 1, Figure S13 for further details.

Assuming *n *= 3 subpopulations - normal cells plus two distinct subpopulations of cancer cells - we analyzed a subset of longer intervals that are most informative for copy number analysis (Table 3). THetA's estimate of 72% tumor purity is slightly higher than the 70% reported by [25]. Moreover, THetA's estimate of tumor purity is higher than the approximately 65% to 67% tumor purity given above for ABSOLUTE, ASCAT and CNAnorm, three methods that assume only one tumor subpopulation. Our BIC model selection chose this solution as a better representation of the data (Figure 3A), than the solution that only considers a mixture of normal cells and a single tumor population. Using the *n *= 3 model we identified all copy number variants identified above for a single tumor population, plus some additional aberrations including subclonal deletions of chromosomes 8, 11, 12, 14 and 15 not identified under that model (nor by the other algorithms). This demonstrates THetA's ability to identify copy number aberrations in subpopulations of cells. While many of the clonal and subclonal copy number aberrations found by THetA are identical to those reported by [25], there are several notable differences including: a clonal deletion of 16q and different classification of aberrations on chromosomes 1 and 22 as clonal vs. subclonal. Table 3 displays the complete set of differences where aberrations in bold indicate a difference between our predictions and those reported by [25]. Aberrations reported by [25] include several chromosomes not considered as part of our analysis (2, 6, 7, 9, 18 and 21). In Table 3 italicized aberrations were not input to the *n *= 3 THetA analysis, and were inferred by examination of read depth ratios corrected for normal admixture and tumor cell fractions derived from THetA (see Additional file 1, Section Q).

We investigated further the following three differences between our analysis and [25]: (1) clonal deletion of chromosome 16q, (2) clonal vs. subclonal amplification of chromosome 1q and (3) clonal vs. subclonal deletions in chromosome 22q. We analyzed these differences using two complementary approaches. First, we analyzed the distribution of tumor/normal read depth ratios in 50 kb bins across the genome. This distribution contains distinct peaks corresponding to copy number aberrations occurring in different subpopulations. After correcting the read depth ratios for a normal admixture using a linear scaling (see Additional file 1, Section O), peaks corresponding to clonal aberrations will occur at ratios divisible by 0.5, whereas peaks corresponding to subclonal aberrations will not (Figure 3B). Second, we analyzed a virtual SNP array that we constructed from the read counts and the variant allele frequencies derived from aligned reads at known germline SNPs (see Materials and methods). Copy number aberrations occurring in different subpopulations appear as distinct clusters in a scatter plot of read count vs. variant allele frequencies (Figure 3C).

The first difference we analyzed was our prediction of a clonal deletion of chromosome 16q, which was not reported by [25]. Visual inspection of the virtual SNP array data for chromosome 4 (predicted to be a clonal deletion by both methods) and chromosome 16q shows three distinct clusters - one for regions of normal copy (centered at a variant allele frequency of 0.5) and two clusters (positioned symmetrically around a variant allele frequency of 0.5) with a lower read count that indicate a deletion (Figure 3D). These deletion clusters have virtually identical locations in the scatter plot for chromosomes 4 and 16q - supporting the conclusion that these deletions occur in the same fraction of the tumor sample. Comparing the difference between the observed and expected read depth ratios in these deletions for different aberration fractions (the percentage of the sample containing the aberration) reveals that the optimal aberration fraction for both deletions is very similar - additional evidence that these deletions occur in the same fraction of the tumor sample (see Additional file 1, Section Q and Figure S12). Given the strong evidence for this chromosome 16 deletion, we suspect that its omission from [25] was an oversight rather than a deficiency of the analysis.

The second difference is that we predicted chromosome 1q to be amplified in a subclonal population consisting of 61.9% of the cells in the sample, whereas [25] indicated that this aberration is clonal (occurring in 70% of cells in the sample). Since this was the only large amplification present in the sample, we were not able to compare its variant allele frequencies to a different amplification (as we did with chromosomes 4 and 16 above). Therefore, we examined the read depth data more closely. Visual inspection of read depth ratios after adjusting for our predicted 28% normal admixture (Figure 3B) and the 30% normal admixture predicted by [25] (see Additional file 1, Figure S11) shows that the corrected read depth ratios for chromosome 1q do not match a ratio of 1.5 well (as would be expected if there was a clonal amplification with copy number 3) - an indication that 1q is a subclonal aberration. Comparison of read depth ratios for 1q to other clonal aberrations supports our prediction that 1q is a subclonal deletion (see Additional file 1, Figure S12).

The final difference involves chromosome 22q; we predicted that it contains both clonal and subclonal deletions, while [25] only reported subclonal events. In particular, [25] reported that a deletion of a derivative chromosome from a translocation between chromosomes 1 and 22 is the rearrangement responsible for the subclonal deletion observed on 22q. We found that 1p (see Additional file 1, Figure S12) and the distal portion of 22q (cytogenetic bands 12.2-13.3) appear to be clonal deletions, while the proximal portion of 22q (cytogenetic bands 11.2-12.1) is a subclonal deletion. In particular, the read-depth/variant-allele plot from the virtual SNP array shows an oblong cluster for chromosome 22 that only partially overlaps with the cluster for chromosome 13, a chromosome predicted by both methods to have undergone a subclonal deletion (Figure 3E). This evidence supports another possible sequence of rearrangements: (1) A non-reciprocal translocation occurred between chromosomes 1 and 22 (supported by the output from the GASV algorithm [48] for clustering of discordant reads as discussed in Additional file 1, Section Q) resulting in the clonal loss of 1p and 22q12.2-13.3. Following this translocation, two copies of 22q11.2-12.1 remained. (2) One of these remaining two copies of 22q11.2-12.1 was deleted in a subclonal population (see Additional file 1, Figure S13).

#### Breast tumor: 40× sequence coverage

We also analyzed two tumor samples from [25] sequenced at approximately 40× coverage. For sample PD4088a, the model of this mixture preferred by our model selection procedure was a single clonal tumor population with normal admixture fraction 41%. [25] also reported this sample as clonal, although they did not provide an estimate of tumor purity or copy number aberrations. Further details of the analysis of this sample are in Additional file 1, Section S and Figure S16.

**Figure 4:**
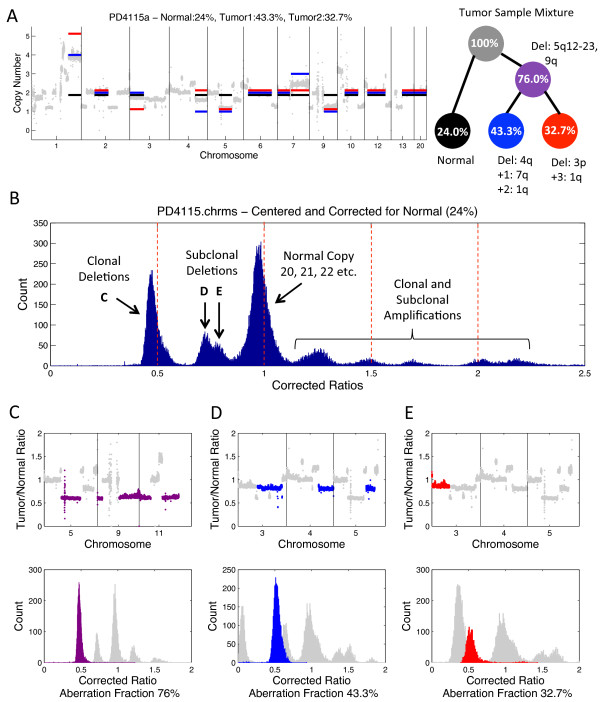
**Analysis of the 40× coverage breast tumor PD4115a**. **(A) **(Left) Read depth ratios (gray) and the inferred copy number aberrations calculated by our algorithm when *n *= 3 including the normal population (black), dominant (clonal) tumor population (blue) and subclonal tumor population (red). (Right) A reconstruction of the tumor mixture with the inferred aberrations and estimated fraction of cells in each subpopulation. **(B) **Distribution of read depth ratios over 50 kb intervals after centering and correction for 24% normal admixture using a simple linear scaling. Several peaks fall near to expected corrected ratios (0.5, 1, 1.5, 2). Two overlapping but distinct peaks can be seen indicating multiple subclonal deletions in similar proportions (labeled D and E). **(C) **(Top) Read depth ratios in 50 kb bins for chromosomes 5, 9 and 11, each of which has a clonal deletion (purple). (Bottom) Distribution of read depth ratios after correction for the aberration fraction of 76% of the sample. **(D) **(Top) Read depth ratios in 50 kb bins for chromosomes 3, 4 and 5, each of which has a subclonal deletion (blue). (Bottom) Distribution of read depth ratios after correction for the aberration fraction of 43.3% of the sample. **(E) **(Top) Read depth ratios as in (D), but a different subclonal deletion is highlighted (red). (Bottom) Distribution of read depth ratios after correction for the aberration fraction of 32.7% of the sample.

We analyzed sample PD4115a, sequenced at approximately 40× coverage using THetA, again considering the case where the mixture contains a normal admixture with a single tumor subpopulation (*n *= 2) and a normal admixture with two distinct tumor subpopulations (*n *= 3). Our BIC model selection chose the model where the sample is a mixture of normal cells and two distinct subpopulations of tumor cells (Figure 4A) over the model where the sample contains a single tumor subpopulation with a normal admixture. While [25] provided some analysis of aberrations in this example, they did not provide a complete tumor history as they did for the 188× coverage genome. Complete information of our analysis of this sample, when we consider it as a mixture of a single tumor subpopulation along with a normal admixture, is in Additional file 1, Section R and Figure S14. For the model considering multiple tumor subpopulations, we analyzed a subset of longer intervals that are most informative for copy number analysis (further details are in Materials and methods). We estimated a normal admixture of 24% (tumor purity 76%) and two tumor subpopulations of 43.3% and 32.7%. The presence of these subclonal populations is apparent from visual inspection of corrected read depth ratios after centering the distribution (ratios in chromosome 20 - which is predicted to contain no copy number aberrations - are translated to have a mean ratio of 1) and correction for a normal admixture (Figure 4B). In particular, a large peak near a corrected ratio of 0.5 represents clonal deletions (Figure 4C). In addition, two overlapping, but distinct smaller peaks appear between the clonal deletions and regions of normal copy (Figure 4D and 4E). These peaks represent two distinct subclonal populations in the tumor sample. A statistical test of the difference in read depth ratios between these peaks supports the conclusion that these subclonal populations are indeed distinct (see Additional file 1, Figure S15).

**Figure 5:**
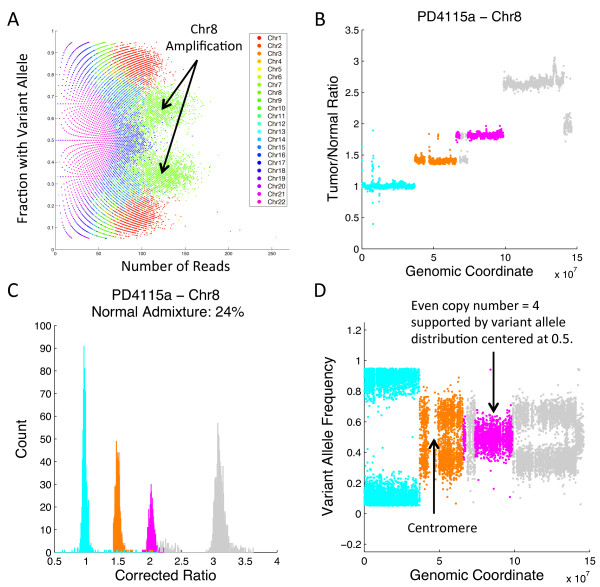
**Analysis of chromosome 8 in sample PD4115a**. **(A) **Virtual SNP array data from this sample show few distinct clusters (compared with the 188× sample in Figure 3A), with amplification of chromosome 8 (green) being the most prominent. **(B) **Read depth ratios for chromosome 8 organized by genomic coordinate. **(C) **Histograms of read depth ratios for chromosome 8 corrected for 24% normal admixture, indicating regions of copy numbers 2, 3 and 4 (cyan, orange and pink), with the latter two being clonal amplifications. **(D) **Variant allele frequencies for chromosome 8. The region with copy number 4 (pink) has variant allele frequencies clustered around 0.5, suggesting duplication of both chromosomal homologs, while the telomeric region with copy number 2 (cyan) has a loss of heterozygosity, suggesting a copy neutral LOH event. LOH: loss of heterozygosity

Virtual SNP array analysis of this sample was difficult due to the lower sequence coverage. This leads to overlapping clusters in the read-count/variant-allele plot, as well as distinct banding resulting from the integrality of read counts (Figure 5A). The only clearly distinct clusters are for highly amplified regions, which have correspondingly higher read counts. Since our analysis for this model used only a subset of chromosome intervals to infer normal admixture and tumor subpopulations, we were able to use the resulting genome mixing vector to analyze other chromosomes that were not used in computing the maximum likelihood solution. We analyzed several regions in chromosome 8 (Figure 5B), a chromosome with a complicated amplification pattern. After correcting read depth ratios in 50 kb intervals in this region for the estimated normal admixture of 24%, three distinct peaks centered near ratios of 1, 1.5 and 2 were apparent, corresponding to integer copy numbers of 2, 3 and 4, respectively, in the tumor sample (Figure 5C). The amplifications are clonal aberrations. Interestingly, the variant allele frequencies for germline SNPs in the regions corresponding to the peak at corrected ratio 2 (copy number 4) are centered at 0.5. This implies that both homologs of chr8q13-21 are present at equal copy number in this region; that is, there is a duplication of both homologs (Figure 5D). In addition, we observed that the variant allele frequencies for chromosome 8p are centered at the values of 0 and 1, although this segment of the chromosome is inferred to have copy number 2. This indicates that there was a copy-neutral loss of heterozygosity (LOH) in this region. LOH in 8p has been previously reported in breast cancer [49,50] and copy neutral LOH in 8p has been reported in cell line data for other cancers [51].

## Discussion

We introduce Tumor Heterogeneity Analysis (THetA), an algorithm that infers the most likely collection of genomes and their proportions from high-throughput DNA sequencing data, in the case where copy number aberrations distinguish subpopulations. We show that THetA outperforms three other methods, CNAnorm [18], ASCAT [12] and ABSOLUTE [13], for inferring tumor purity and identifying copy number aberrations in the case of a single tumor cell population admixed with normal (non-cancerous) cells. Moreover, we demonstrate that THetA successfully estimates tumor purity even at low purity (10%) and with modest sequence coverages (approximately 30×) on both real and simulated data. In contrast to other recent methods [12,13] that first infer average ploidy across the genome, THetA simultaneously estimates tumor purity and computes the integral copy number of each genomic segment/interval. These advantages result from THetA exploiting the large number of data points (reads) that measure copy number aberrations in high-throughput sequencing data - information that is not available from SNP arrays.

We also demonstrate that THetA successfully deconvolves a tumor sample into a normal population and multiple tumor subpopulations, inferring the proportion of each subpopulation in the mixture, and partitioning copy number aberrations into clonal and subclonal populations. Other existing methods, such as ASCAT [12], ABSOLUTE [13] and CNAnorm [18], do not directly infer multiple subpopulations. Further, we show that these methods can produce highly inaccurate estimates of tumor purity on samples containing multiple subpopulations, and are sometimes unable to identify some copy number aberrations that occur in subpopulations of tumor cells. In addition, THetA reports all possible solutions of interval count matrices **C **and genome mixing vectors *μ *with the same maximum likelihood, allowing users to explore different maximum likelihood solutions. Thus, THetA is an attractive alternative to these methods.

We demonstrated the advantages of THetA using three breast cancer genomes sequenced in [25]: one sequenced at approximately 188× coverage and two at approximately 40× coverage. Nik-Zainal *et al. *[25] showed how a large amount of information about a tumor's evolutional history can be derived by analyzing clonal and subclonal mutations in high-coverage sequencing data. Our THetA algorithm automates some of the manual analysis involved in such reconstructions. For the 188× genome, our results are largely concordant with the extensive analysis and annotation of this sample in [25]. THetA automatically recovered nearly all of the copy number aberrations reported in [25], but with some differences in the classification of aberrations as clonal or subclonal. Allele data not used by THetA provides external evidence that support the THetA results in several cases. On one of the 40× coverage genomes, we identified two previously unreported tumor subpopulations in nearly equal proportions, as well as a 24% normal admixture. These results are supported by statistical comparisons of read depth ratios, and also allowed us to identify copy-neutral LOH on chromosome 8q. Thus, we demonstrated that it is possible to identify multiple tumor populations successfully in a single sample by considering a subset of genomic intervals. Further, we did so for an approximately 40× sequenced tumor, demonstrating the ability to identify intra-tumor heterogeneity at sequence coverages that are the current standard in cancer sequencing studies.

THetA uses only read depth for inferring intra-tumor heterogeneity, in contrast to other methods [12,13,17,25] that use allele frequencies of heterozygous germline SNPs and somatic mutations. Since copy number aberrations - even those of a modest size - affect a large number of reads, THetA is able to infer multiple tumor subpopulations directly from sequencing data. However, THetA also has some limitations. First, the reliance on copy number aberrations means that THetA is unable to identify tumor subpopulations that do not contain copy number aberrations. As copy number aberrations are ubiquitous in many types of cancers, particularly solid tumors, we expect that THetA will prove useful for analyzing a wide range of different cancer samples. Second, while the mathematical model used by THetA allows for any number of subpopulations, in practice the number of subpopulations that can be correctly inferred depends on having at least one copy number aberration that distinguishes every subpopulation. Finally, THetA's computation time increases with an increasing number of subpopulations.

Our focus in the development of THetA was to address rigorously the difficult problem of analyzing tumor purity and subclonal copy number aberrations from DNA sequencing data. A logical next step is to use the output from THetA to help predict single-nucleotide mutations in tumor samples and/or assess the clonality of somatic mutations, both challenging problems in their own right. Carter *et al. *[13] and Nik-Zainal *et al. *[25] show that once tumor purity is correctly estimated, then this value can be used to analyze the clonality/subclonality of somatic mutations. Incorporating the additional signal of variant allele frequencies into the probabilistic model, as well as extending the model to allele-specific copy number changes [52], are important directions for future work. Ultimately, a desirable goal is to integrate into a single probabilistic framework the detection of all types of somatic aberrations (single nucleotide, copy number and rearrangements) with the estimation of tumor purity and the derivation of tumor subpopulations. Finally, further algorithmic improvements in THetA would help in the analysis of more complicated tumor samples that have more intervals (for example, smaller copy number aberrations), higher amplitude copy number aberrations, more subpopulations or more complicated rearrangements; for example, due to breakage/fusion/bridge (B/F/B) cycles [53], chromothripsis [54] or extrachromosomal amplifications [55]. THetA runs in polynomial time for a mixture of two genomes with intervals of equal weight, but the question of the complexity of the MLMDP for *n *> 2 remains open.

A number of other techniques have recently been used to study intra-tumor heterogeneity. For example [56] uses expression profiles across different individuals to identify differentially expressed genes with respect to healthy cells at the cancer site of origin. Single-cell sequencing and multi-region sequencing from a primary tumor are alternative strategies that have been successfully employed [23-29]. As these technologies improve they will likely further contribute to our understanding of intra-tumor heterogeneity. However, sequencing of primary tumor samples as well as matched tumor/metastasis samples will remain a dominant protocol for some time. Thus, algorithms, such as THetA, ABSOLUTE, ASCAT and others, that can derive information about intra-tumor heterogeneity from DNA sequencing of tumor samples are a useful complement to other technologies and techniques for tumor heterogeneity studies.

## Conclusions

Tumors are highly heterogeneous with individual cells in a tumor typically having different complements of somatic mutations. Highly accurate estimates of tumor purity and tumor subpopulation frequencies are necessary for investigating intra-tumor heterogeneity from single tumor samples. We introduce THetA, an algorithm that infers the most likely collection of genomes and their proportions from high-throughput DNA sequencing data, in the case where copy number aberrations distinguish subpopulations. We show the power of THetA with both simulated and real sequencing data - demonstrating the ability to identify intra-tumor heterogeneity (in particular subclonal copy number aberrations) at modest sequence coverages (approximately 40×) that are the current standard in cancer sequencing studies.

## Materials and methods

### Intervals and counts: probability model

In this section we derive the probability *P*(**r**|**C**, *μ*) in the maximum likelihood mixture decomposition problem (MLMDP).

#### Single genome

Following the usual assumptions (for example, the Lander-Waterman model), we assume that the starting positions of reads in a cancer genome are uniformly distributed over its length. The probability of a read from a cancer genome aligning to an interval *I_j _*in the reference genome depends on: (i) the number of copies of the interval in the cancer genome, (ii) the length of the interval and (iii) possible difficulties in aligning reads to *I_j _*due to repetitive sequence or other effects. We first describe the model under the simplifying assumption that there are no alignment difficulties and all the intervals in **I **are of equal length, which without loss of generality we set to length 1. Below we show how to remove these restrictions by incorporating an interval weight vector **w **into the model, which assigns a weight to each interval in proportion to its length or mappability. Let **c **= (*c*_l_, ..., *c_m_*) be the (unknown) number of copies of each interval in the cancer genome. Then the probability *p_j _*that a read aligns to *I_j _*satisfies:

$$p_{j}=\frac{c_{j}}{{\left|c\right|}_{1}}$$

where ${\left|c\right|}_{1}=\sum_{j=1}^{m}c_{j}$ is the *l*_1_-norm of **c**. We use the notation:

$$\hat{x}=\frac{x}{{\left|x\right|}_{1}}$$

to denote a normalized vector. Thus, the observed read depth vector **r **is the result of $r=\sum_{j=1}^{m}r_{j}$ independent draws from a multinomial distribution with parameter $p=\hat{c}$; that is, $r\sim \text{Mult}\left(\text{r;}\hat{c}\right)$.

We emphasize that the number of reads aligning to each interval are not independent random variables. As the total number of reads becomes extremely large, the multinomial distributions will converge to independent Poisson distributions in each interval. However, even with the millions to billions of reads produced by high-throughput DNA sequencing, the effects of using a finite number of reads are an issue in cancer genome sequencing. This is because large copy number changes - including the gain and loss of whole chromosomes - are common in cancer genomes. A large deletion or duplication will affect the number of reads observed in other intervals; for example, if we consider the 22 autosomes, a homozygous deletion of chromosome 1 will reduce the effective length of the cancer genome by 8.65%, altering the expected number of reads observed in other intervals (see Additional file 1, Figure S1).

#### Mixture of genomes

Now suppose we sequence a tumor sample  and align the obtained reads to the reference genome, observing a read depth vector **r **= (*r*_1_, ..., *r_m_*) ∈ ℕ*^m^*. Let **C **= [*c_jh_*] be the (unknown) interval count matrix and *μ *be the (unknown) genome mixing vector for the tumor sample. Here *μ *is required to be an element of the unit (*n *- 1)-simplex $\Delta_{n-1}=\left\{{\left(\mu_{1},\ldots ,\mu_{n}\right)}^{\text{T}}\in {\mathbb{R}}^{n}|\overset{n}{\underset{h=1}{\sum }}\mu_{h}=1,\text{ and }\mu_{h}\geq 0\text{ for all }h\right\}$. Then the probability *p_j _*that a read aligns to *I_j _*is the ratio of DNA in  from *I_j _*compared to the total amount of DNA in the sample. That is:

$$p_{j}=\frac{{\left(C\mu \right)}_{j}}{{\left|C\mu \right|}_{1}}$$

Therefore, **r **is the result of $r=\sum_{j=1}^{m}r_{j}$ draws from a multinomial distribution with parameter (Figure 1):

$$p=\hat{C\mu }=\frac{C\mu }{{\left|C\mu \right|}_{1}}$$

That is $r\sim \text{ Mult}\left(r;\hat{C\mu }\right)$.

### Solving the maximum likelihood mixture decomposition problem

We show here how to solve the MLMDP as a disjunction of separate convex optimization problems. The negative log-likelihood of **r **as a function of the generic multinomial parameter $p\in {\text{Δ}}_{m-1}$ is:

(1) $${ℒ}_{r}\left(p\right)=-\text{log}\left(P\left(r|p\right)\right)=-\text{log}\left(\frac{\left(\overset{m}{\underset{j=1}{\sum }}r_{j}\right)!}{\overset{m}{\underset{j=1}{\prod }}r_{j}!}\overset{m}{\underset{j=1}{\prod }}{\left(p_{j}\right)}^{r_{j}}\right)=-\overset{m}{\underset{i=1}{\sum }}r_{i}\text{log}\left(p_{i}\right)+\alpha$$

where α is a constant, depending only on **r**. Finding the multinomial parameter **p **that minimizes this negative log-likelihood function is straightforward. Using a Lagrange multiplier to encode the constraint $p\in {\text{Δ}}_{m-1}$, one determines that the (unique) value p* maximizing ${ℒ}_{r}\left(p\right)$ satisfies:

$$p_{i}^{*}=\frac{r_{i}}{\overset{m}{\underset{j=1}{\sum }}r_{j}}$$

Moreover, if the entries of **r **are integers (as they will be for read counts) and **C **is permitted to be any integer-valued matrix, then the (unconstrained) solution:

$$p_{i}^{*}=\frac{r_{i}}{\overset{m}{\underset{j=1}{\sum }}r_{j}}$$

can be written in the form $p=\hat{C\mu }$ (see Additional file 1, Section C). Thus, a solution of the MLMDP is obtained by maximizing the multinomial likelihood over all $p\in {\text{Δ}}_{m-1}$.

#### Constraints on C

In the Results section above, we described three natural constraints on the interval count matrix **C**. We define Ω*_m,n _*to be pairs (**C**, *μ*) where **C **satisfies the first two of those conditions:

(2) $${\text{Ω}}_{m,n}=\left\{\left(C,\mu \right)|c_{1}=2^{m},c_{j}\in {\mathbb{N}}^{m}\text{ for }j>1,\mu \in {\text{Δ}}_{n-1}\right\}$$

Similarly, we define Ω*_m,n,k _*⊆ Ω*_m,n _*to be the pairs (**C**, *μ*) where **C **satisfies all three of the conditions:

(3) $${\text{Ω}}_{m,n,k}=\left\{\left(C,\mu \right)|c_{1}=2^{m},c_{j}\in {\left\{0,\ldots ,k\right\}}^{m}\text{ for }j>1,\mu \in {\text{Δ}}_{n-1}\right\}$$

In the following, we will use Ω to refer to either Ω*_m,n _*or Ω*_m,n,k_*, as appropriate. Given a pair (**C**, *μ*) ∈ Ω, we define the negative log-likelihood of the observed read depth vector **r **using the multinomial model to be:

(4) $${ℒ}_{r}\left(C,\mu \right)=-\text{log}\left(P\left(r|C,\mu \right)\right)=-\overset{m}{\underset{i=1}{\sum }}r_{i}\text{log}\left({\left(\hat{C\mu }\right)}_{i}\right)+\alpha$$

For an observed **r**, our goal is to find the **C **and *μ *that minimize (4). We define the following optimization problem where the domain of (**C**, *μ*) can be either of the domains Ω defined above:

(5) $$\left(C^{*},\mu^{*}\right)=\text{argmi}{\text{n}}_{\left(C,\mu \right)\in \text{Ω}}{ℒ}_{r}\left(C,\mu \right)=\text{argmi}{\text{n}}_{\left(C,\mu \right)\in \text{Ω}}{ℒ}_{r}\left(\hat{C\mu }\right)$$

Since all entries of **C **are positive integers and all *μ_j _*are positive reals, (5) is a mixed integer problem. In general, mixed integer linear programming (MILP) problems are NP-hard to solve [57]. In our case, the objective function is a non-linear function of **C **and *μ*, meaning that even sophisticated MILP solvers are unlikely to be much benefit for this problem.

#### A coordinate transformation

Rather than attempting to solve the optimization problem (5) as a generic MILP, we derive a coordinate transformation that allows us to solve this problem as a constrained optimization problem in ℝ*^m^*. First, note that a pair (**C**, *μ*) ∈ Ω defines a probability distribution $\hat{C\mu }$. We define $P_{\text{Ω}}=\left\{\hat{C\mu }|\left(C,\mu \right)\in \text{Ω}\right\}$ to be the space of all such probability distributions for all (**C**, *μ*) ∈ Ω. Note that only $\hat{C\mu }$, and not (**C**, *μ*), is identifiable from the observed data **r**. We prove the following theorem in Additional file 1, Section D.

**Theorem 1**. *Suppose ***p ***∈ P*_Ω_, *so *$\text{p = }\hat{C\mu }$, *for some *(**C***, μ*) ∈ Ω*. Then there exists μ' ∈ *Δ*_n - 1 _such that $p=\hat{C}\mu^{\prime }$ where $\hat{C}=\left(\hat{c_{1}},\ldots ,\hat{c_{n}}\right)$*.

Now suppose the interval count matrix **C **is fixed, and let $H\left(C\right)=\left\{\hat{C}\mu |\mu \in {\text{Δ}}_{n-1}\right\}$ denote the set of convex combinations of the normalized column vectors in **C**. Then (5) reduces to the problem of finding argmin _**p **∈ *H*(**C**) _${ℒ}_{r}\left(p\right)$. Since the objective function ${ℒ}_{r}\left(p\right)$ is separable convex (see Additional file 1, Section F) and the domain *H*(**C**) is convex, this problem is easy to solve using standard convex optimization routines.

Let $C_{m,n,k}=\left\{C|\left(C,\mu \right)\in {\text{Ω}}_{m,n,k}\right\}$ be the set of interval count matrices **C **appearing in Ω*_m,n,k_*. Considering all interval count matrices $\text{C}\in C_{m,n,k}$ gives the following optimization problem:

(6) $$\text{min}{ℒ}_{r}\left(p\right)\text{ subject to }p\in \cup_{C\in C_{m,n,k}}H\left(C\right)$$

**Figure 6:**
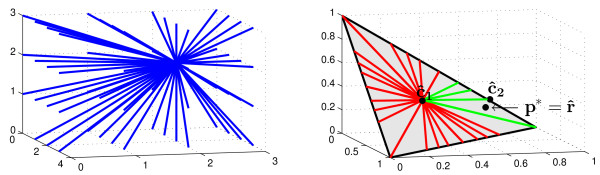
**Convex geometry of the MLMDP used in the THetA algorithm**. (Left) For a single cancer genome with normal admixture, the interval count vector **c**_2 _of the cancer genome and tumor purity *μ *define a collection of rays **C***μ*, for *μ *∈ 0[1]. (Here we show the space Ω_3,2,3_). (Right) Normalizing these rays, we obtain the parameter $p=\hat{C\mu }$, used in the multinomial likelihood. These parameters are embedded in the simplex Δ*_m _*_- l_(gray triangle with a black outline) because their entries sum to one. (This is the space $P_{{\text{Ω}}_{3,2,3}}$.) For a fixed interval count matrix **C **= (**c**_1_, **c**_2_) a blue ray (left) defined by **C***μ *is mapped to the corresponding red/green ray (right) connecting $\hat{c_{1}}$ to $\hat{c_{2}}$ (right), the normalized columns of **C**, as described in Theorem 1. For *n *> 2, hyperplanes are mapped to hyperplanes (see Additional file 1, Figure S2). We show $p^{*}=\hat{r}$, the maximum likelihood solution when interval counts are not constrained to be integers. Note that this point is not on any of the rays defined by interval count matrices. Rays that satisfy the ordering constraint from Theorem 2 are in green. MLMDP: maximum likelihood mixture decomposition problem

Figure 6 illustrates the geometry of this optimization problem. Since in general a union of convex sets is not convex, the constraint set in (6) is not convex. A brute-force approach to this problem is to enumerate all **C **∈ $\hat{C\mu }$, but the number of such matrices is exponential in *m *and *n*. Note that in the Results section, THetA demonstrates improved performance in computing **C **and *μ *when the number *m *of intervals increases in the case where *n *= 3. This is expected from the convex geometry used by our algorithm: for a fixed interval count matrix **C**, each value $\hat{C\mu }$, defines a 2-plane in Δ_*m *- 1 _(see Additional file 1, Figure S2). These planes become more sparse in Δ_*m *- 1 _as *m *increases, and thus our algorithm is less prone to overfitting. In the next section, we show that in the *n *= 2 case we can restrict the space of **C **matrices to a number that is polynomial in *m*.

### A more efficient algorithm for the MLMDP

We derive an algorithm to solve the MLMDP (as formulated in (6)) that is polynomial time in *m *when *n *= 2. This algorithm relies on the observation that it is necessary to consider only a subset of interval count matrices **C **whose entries satisfy ordering constraints imposed by the read depth vector **r**. We say that two vectors **a **= (*a*_1_, ..., *a_m_*) and **b **= (*b*_1_, ..., *b_m_*) ∈ ℝ*^m ^*have compatible order if for all 1 ≤ *i*, *j *≤ *m*, *a_i _*≤ *a_j _*if and only if *b_i _*≤ *b_j_*. Note that the vector **x **= (*s*, ..., *s*) ∈ ℝ*^m ^*for any *s *∈ ℝ has compatible order with all vectors in ℝ*^m^*.

**Theorem 2**. Suppose $p^{*}=\hat{C^{*}\mu^{*}}=\text{argmi}{\text{n}}_{p\in P_{{\text{Ω}}_{m,n,k}}}{ℒ}_{r}\left(p\right)$. Then we have the following:

*1*. p* and **r ***have compatible order*.

2. If n = 2 and μ_2_^* ^> 0, then **r ***and ***c**_2_^* ^have compatible order.

Theorem 2 (proof is in Additional file 1, Section E) leads to a more efficient algorithm where we evaluate only matrices ${\text{C = (c}}_{1},{\text{c}}_{2})\in C_{m,2,k}$ where **c**_2 _has compatible order with **r**. The number of such matrices is *O*(*m^k^*) and enumeration of these matrices is *O*(*m*^*k*+1^) (see Additional file 1, Section E). Note that if *n *≥ 3, that is, there is more than one cancer genome in the mixture, then the ordering constraints do provide some restrictions on the entries of **C **(see Additional file 1, Section E). The reduction is not enough to make the space have polynomial size (in *m*), but the restrictions are useful in practice.

### Intervals of unequal length and mappability

Thus far, we made the simplifying assumptions that all intervals in **I **are of equal length and that reads are aligned to each interval without any biases from the DNA sequence of the interval. Now we consider the general case where each interval *I_j _*has an associated positive weight *w_j_*. These weights can model both interval lengths as well as different mappability of intervals - that is, the probability of reads aligning uniquely to an interval in the reference genome can depend on the repeat content of the interval [58]. Let **w **= (*w*_1_, ..., *w_m_*) be the interval weight vector. In practice, we use the read depth vector over **I **for the paired normal sample as **w**, which allows us to implicitly incorporate information on interval length, mappability and GC content into the model.

Consider a single cancer genome where **c **= (*c*_1_, ..., *c_m_*) is the number of copies of each interval in the cancer genome. Then the probability *p_j _*of a read aligning to interval *I_j _*in the reference genome is:

$$\frac{w_{j}c_{j}}{\overset{m}{\underset{i=1}{\sum }}w_{i}c_{i}}=\frac{w_{j}c_{j}}{{\left|Wc\right|}_{1}}$$

where **W **is a diagonal matrix such that *W_j,j _*= *w_j_*. Therefore, the observed read depth vector **r **is obtained by $r=\sum_{j=1}^{m}r_{j}$ independent draws from a multinomial distribution with parameter:

$$p=\left(\frac{w_{1}c_{1}}{{\left|Wc\right|}_{1}},\ldots ,\frac{w_{m}c_{m}}{{\left|Wc\right|}_{1}}\right)$$

We define the linear transformation Φ: ℝ*^m ^*→ ℝ*^m ^*to be $\text{Φ}\left(v\right)\;=\;\hat{Wv}$. Thus, **p **= Φ(**c**) and **r **~ Mult(*r*; Φ(**c**)). As in the unweighted case above, if the entries in **c **are allowed to be arbitrary positive integers, then for any integer read depth vector **r **and non-negative weight vector **w **we can always find the maximum likelihood solution to the corresponding weighted MLMDP (see Additional file 1, Section G).

Similarly, if we consider a tumor mixture  with interval count matrix **C **and genome mixing vector *μ*, the probability *p_j _*of a read aligning to interval *I_j _*satisfies:

$$p_{j}=\frac{w_{j}{\left(C\mu \right)}_{j}}{\overset{m}{\underset{i=1}{\sum }}w_{i}{\left(C\mu \right)}_{i}}=\frac{{\left(WC\mu \right)}_{i}}{{\left|WC\mu \right|}_{1}}=\text{Φ}\left(C\mu \right)$$

Given a read depth vector **r **and an interval weight vector **w**, we formulate the analogous maximum likelihood mixture decomposition problem of identifying the underlying interval count matrix **C **and genome mixing vector *μ *that maximize the multinomial likelihood Mult(**r**|Φ(**C***μ*)).

Theorem 3 (see Additional file 1, Section G for the proof) relates the optimal (**C**, *μ*) in the cases of equal and unequal weighted intervals.

**Theorem 3**. *Let *Φ^-1 ^: ℝ*^m ^*→ ℝ*^m ^be *${\text{Φ}}^{--1}\left(v\right)\;=\hat{W^{-1}v}$. *We have the following set equality:*

$$\text{argmi}{\text{n}}_{\left(C,\mu \right)\in {\text{Ω}}_{m,n}}{ℒ}_{r}\left(\text{Φ}\left(C\mu \right)\right)=\text{argmi}{\text{n}}_{\left(C,\mu \right)\in {\text{Ω}}_{m,n}}{ℒ}_{{\text{Φ}}^{-1}\left(r\right)}\left(\hat{C\mu }\right)$$

Using this theorem, we find the optimal solution in the weighted interval case by solving the unweighted interval case; for example, using the techniques above. As stated, Theorem 3 applies to the case where (**C**, *μ*) ∈ Ω*_m,n _*(that is, the entries of **C **are unbounded). However, we can still leverage the logic behind this result when we add a restriction that $\text{C}\in C_{m,2,k}$. While we do not expect that $\text{argmi}{\text{n}}_{\left(C,\mu \right)\in {\text{Ω}}_{m,2,k}}{ℒ}_{r}\left(\text{Φ}\left(C\mu \right)\right)$ is equal to $\text{argmi}{\text{n}}_{\left(C,\mu \right)\in {\text{Ω}}_{m,2,k}}{ℒ}_{{\text{Φ}}^{-1}\left(r\right)}\left(\hat{C\mu }\right)$, we may assume that a solution to $\text{argmi}{\text{n}}_{\left(C,\mu \right)\in {\text{Ω}}_{m,2,k}}{ℒ}_{r}\left(\text{Φ}\left(C\mu \right)\right)$ will satisfy the same order constraints as ${ℒ}_{{\text{Φ}}^{-1}\left(r\right)}\left(\hat{C\mu }\right)$. Namely, we expect that the optimal solution will have compatible order with Φ^-1^(**r**) (Theorem 2). This is because: (1) the unconstrained optima (when (**C**, *μ*) ∈ Ω*_m,n_*) for the two likelihood functions are equal, (2) the objective function ${ℒ}_{r}\left(p\right)$ is well behaved (separable convex) and (3) the transformation Φ is linear. Thus, the optima in the constrained weighted case cannot deviate too much from the optima in the constrained unweighted case, where the ordering conditions hold. Thus, we need only to consider $\text{C}\in C_{m,2,k}$ where **c**_2 _has compatible order with Φ^-1^(**r**) to find an optimum. We verified this statement empirically over a variety of simulations (see Additional file 1, Section H).

### Model selection

We use the Bayesian information criterion (BIC) to make a selection from different sized models (that is, different values of *n*) and their corresponding sets of maximum likelihood solutions. The standard form of the BIC is -2 log(*L*) + *a *log(*b*) where *L *is the likelihood of a solution, *a *is the number of free parameters in the model and *b *is the number of data points. We add a slight modification to this, which is similar to a modification used by the segmentation algorithm BIC-Seq [32] that allows use of more stringently penalized solutions with more free parameters using a new parameter *γ*. The motivation is that the BIC tends to be too liberal when the model space is large [59] - as is the case here. Values of *γ *above 1 will penalize models that have more distinct tumor populations more strongly. That is, increasing this parameter will more strongly encourage solutions with fewer subpopulations. The default value of *γ *is 10, and was chosen because we expect to recover a small number of distinct subpopulations from sequencing data - thus making penalization of models with more subpopulations attractive. Additionally, changing *γ *in either direction (by up to 4) from this default value yields consistent results on the datasets analyzed. Our modified BIC is -2 log(*L*) + *γa *log(*b*), where *a *= (*m *+ 1)(*n *- 1) and *b *is the total number of reads in the intervals for both the tumor and normal samples. Since we often run the *n *= 3 version of the algorithm on a subset of the intervals used in the *n *= 2 algorithm, we use the following steps to determine which value of *n *to select. (1) Run the algorithm for *n *= 2 and *n *= 3 using the subset of intervals and the lower and upper bounds used for *n *= 3 and obtain respective likelihood values. (2) Compute the modified BIC for both values of *n *and choose the one with the lowest value.

### Sets of maximum likelihood solutions

If (**C**, *μ*), (**C**', *μ*') ∈ Ω such that $\hat{C\mu }=\hat{C^{\prime }\mu^{\prime }}$, then for any observed read depth vector **r**, the likelihood of observing the **r **will be identical between these two solutions. That is, ${ℒ}_{r}\left(\hat{C\mu }\right)={ℒ}_{r}\left(\hat{C^{\prime }\mu^{\prime }}\right)$. By default THetA will always output the complete set of maximum likelihood solutions to the MLMDP given the input parameters (for example, the maximum copy number *k *to consider). However, THetA has several options that allow a user to input additional information, like sample ploidy, which may be known in advance. One option allows a user to supply an expected ploidy for a sample (for example, 4 in the case of a tetraploid genome), and the lower and upper bounds considered for all intervals are rescaled to reflect this expected ploidy. Another option allows a user to set lower and upper bounds on copy numbers directly for all intervals in the genome. In either case, THetA will still output the complete set of maximum likelihood solutions that reflect the options supplied by the user.

### Code availability

The THetA software is available for download from our website [60]. For a copy of the software at the time of publication please see Additional file 2, although we recommend that the latest version of THetA be downloaded from [60].

### Analysis of breast cancer genomes

Here we provide additional details of the analysis of the breast cancer samples.

#### Breast tumor: 188× sequence coverage

For the *n *= 2 analysis of sample PD4120a, we used all genomic intervals derived following BIC-Seq segmentation (*λ *= 100) after removal of all intervals less than 50 kb in length. For the *n *= 3 analysis, we selected a subset of these intervals by choosing: (1) all chromosomes that BIC-Seq partitioned into a single interval and (2) all intervals >22 Mb that were reported as having an abnormal copy number (≠ 2) in the *n *= 2 analysis. We used only the longest such interval per chromosome if the number of such intervals was large. We later added all intervals from chromosome 22 to this subset in order to resolve differences between our results and those presented in [25]. Since the results for both subsets were extremely similar, we present here the results for the larger subset (including chromosome 22). Results for the smaller subset are given in Additional file 1, Figure S9.

#### Breast tumor: 40× sequence coverage

For the *n *= 2 analysis of sample PD4115a, we used all genomic intervals derived from BIC-Seq segmentation (*λ *= 200) after removal of all intervals 50 kb in length. We found that PD4115a contains many apparent copy number aberrations with the segmentation containing 102 intervals (compared to only 69 intervals for sample PD4120a above). In addition, this sample also includes several highly amplified regions, and no chromosome was segmented into a single interval. Thus, we ran THetA for *n *= 3 on a subset of the longest intervals in the BIC-Seq partition, and set lower and upper bounds on the copy number for each interval (see Additional file 1, Section N).

#### Virtual SNP arrays

To compare those of our predictions that differed from those presented in [25], we looked at known germline SNP allele frequencies derived directly from the sequencing data - a virtual SNP array. We emphasize that this data was not used by our THetA algorithm for computing tumor heterogeneity, and therefore this provides independent data for validation. We looked at read coverage and variant allele frequency for the 907,693 SNP positions on the 22 autosomes tested by the Affymetrix 6.0 SNP array (SNP positions and major and minor alleles for hg19 determined using the UCSC genome browser [61]). The read coverage for a SNP position is the number of concordant reads with mapping quality >30 that have an alignment containing either the major or minor allele at the SNP position. The variant allele fraction, or BAF, is the fraction of such reads that contains the minor allele.

## List of abbreviations used

AML: acute myeloid leukemia; BIC: Bayesian information criterion; kb: kilobase; LOH: loss of heterozygosity; Mb: megabase; MILP: mixed integer linear programming; MLMDP: maximum likelihood mixture decomposition problem; SNP: single nucleotide variant; TCGA: The Cancer Genome Atlas; THetA: Tumor Heterogeneity Analysis.

## Competing interests

The authors declare that they have no competing interests.

## Authors' contributions

LO, AM and BJR developed the algorithm; LO and AM implemented the algorithm and LO performed the experiments. LO, AM and BJR wrote the manuscript. All authors read and approved the manuscript.

## Supplementary Material

Additional file 1 **Figures and text describing additional information such as proofs of theorems or additional experimental results**. Click here for file

Additional file 2 **THetA software package at the time of publication**. In general, it is recommended that the latest version of THetA be downloaded from [60]. Click here for file
