# Supplementary material for: THetA: inferring intra-tumor heterogeneity from high-throughput DNA sequencing data
Source: Genome Biol. 2013 Jul 29;14(7):R80. doi: 10.1186/gb-2013-14-7-r80 (PMC4054893; doi:10.1186/gb-2013-14-7-r80)
Supplement: Additional file 1 — Figures and text describing additional information such as proofs of theorems or additional experimental results. [file gb-2013-14-7-r80-S1.PDF]

Supplemental Material for "THetA: Inferring intra-tumor heterogeneity from high-throughput DNA sequencing data"

Layla Oesper, Ahmad Mahmoody, and Benjamin J. Raphael
Department of Computer Science and Center for Computational Molecular Biology, Brown University, Providence, RI
layla@cs.brown.edu, ahmad@cs.brown.edu, braphael@cs.brown.edu

A Motivation for the Multinomial Model

The multinomial model that we use in our likelihood function does not assume that the observed read depths in different intervals are independent. Even though we assume that reads are distributed uniformly on the cancer genome, large copy number aberrations (e.g. gain and loss of whole chromosomes) will cause the observed number of aligned reads in an interval I_j to deviate from expected *even* when the interval I_j itself is not affected by a copy number aberration. The reason is because the number of reads is fixed; thus, for example the lack of reads aligning to one part of the genome due to a deletion will mean that there will be more reads observed from the non-deleted parts of the genome. We see such instances in the breast cancer sequencing data from [5]. In particular, we see instances where large amplifications or deletions cause tumor/normal read depth ratios for genomic intervals having normal copy 2 are no longer centered at the expected ratio of 1. For 19 tumor/normal pairs, we re-scaled the read depth vector for the normal sample to have the same number of reads as the read depth vector for the tumor sample and looked at the $\log_2$ of the distribution of read depth ratios in the set of intervals returned by running BIC-Seq [10]. In several of these distributions, we see that the mode of the distribution is not centered at 0, as would be expected if the majority of the tumor genome exhibited the same copy number as the normal genome, but is instead shifted slightly to the left or right (see Fig. S1). This shift is a possible indication that the cancer genome has changed significantly in length compared to the normal genome. Thus, considering all intervals independently may lead to incorrect interpretation of intervals as being amplified or deleted depending on the extent of the shift, whereas, our multinomial model takes changes in genome length into account. For example, the distribution for sample PD4248 has its peak shifted to the left (Fig. S1).

B Derivation of Equations Used by ASCAT and ABSOLUTE

In this section we show how Equation 1 from [2] and the log term in Equation 1 from [9] can be written as a function of expected values of observations in our probabilistic model. Let $\mathbf{I} = (I_1, \ldots, I_m)$ be a partition of the reference genome into m intervals. Using our notation, these equations can be written directly as $\frac{2\mu_1 + c_{j2}\mu_2}{2\mu_1 + \rho\mu_2}$ where ρ is average ploidy in the cancer genome. Suppose we sequence a tumor sample $\mathcal{T}$ with P reads. Let X_{pq} be a random variable such that $X_{pq} = 1$ if the pth read from $\mathcal{T}$ aligns to I_q and 0 otherwise. Therefore $X_q = \sum_{n=1}^{N} X_{pq}$ is the number of reads from $\mathcal{T}$ that align to I_q. Let Y_q be a similar random variable, but for a matched normal sample $\mathcal{N}$. We can now calculate the expected number of reads aligning

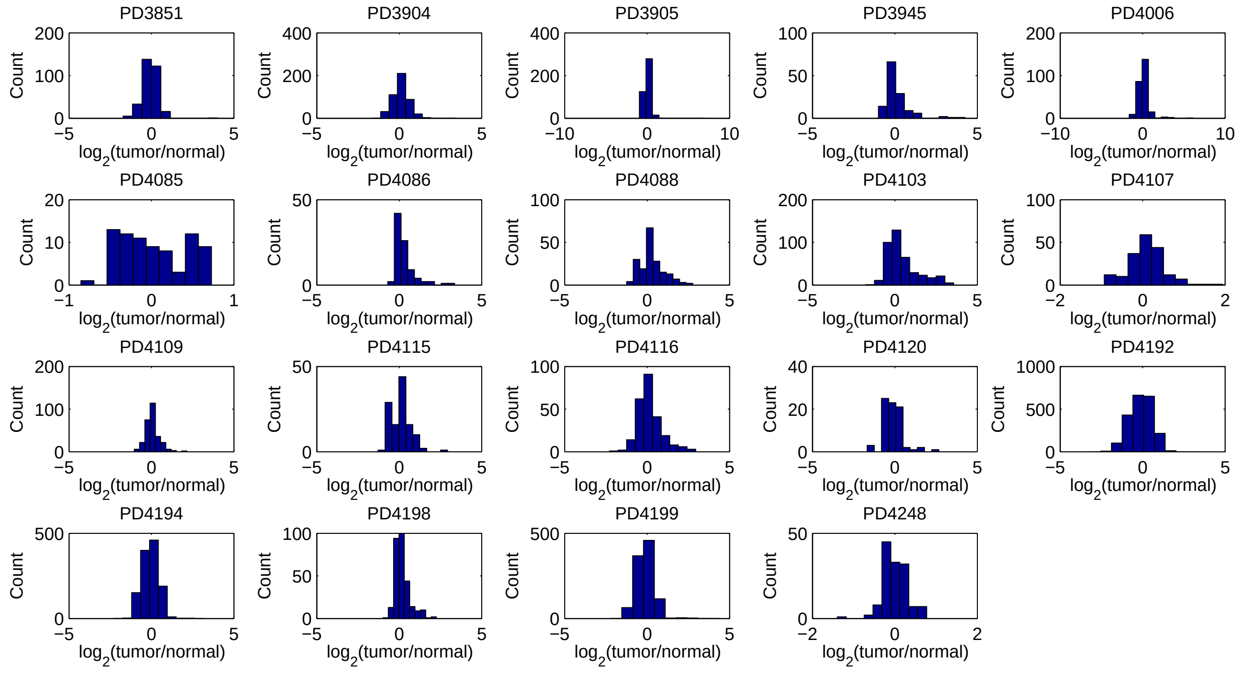

Figure S1: The distribution of $\log_2$ ratios of tumor to normal read depth for 19 breast cancer samples from [5]. Intervals were determined using BIC-Seq with $\lambda = 100$.

to interval I_q.

$$E[X_q] = E[\sum_{p=1}^{P} X_{pq}] = \sum_{p=1}^{P} E[X_{pq}] = \sum_{p=1}^{P} Prob(X_{pq} = 1) = P \times Prob(X_{pq} = 1)$$

Now assume that $\mathcal{T}$ has a true underlying $\mathbf{C}$ and μ where $n = 2$ and the first column of $\mathbf{C}$ is set to 2 (the normal component). Let $\rho = \frac{1}{m}\sum_{k=1}^{m} c_{k2}$, that is ρ is the average value of entries in the second column of $\mathbf{C}$. Using our multinomial model, we can directly calculate $Prob(X_{pq} = 1)$.

$$\begin{aligned} Prob(X_{pq} = 1) = (\widehat{\mathbf{C}\mu})_q &= \frac{(\mathbf{C}\mu)_q}{\sum_{k=1}^{m}(\mathbf{C}\mu)_k} = \frac{2\mu_1 + c_{q2}\mu_2}{\sum_{k=1}^{m}(2\mu_1 + c_{k2}\mu_2)} \\ &= \frac{2\mu_1 + c_{q2}\mu_2}{2m\mu_1 + \mu_2\sum_{k=1}^{m} c_{k2}} = \frac{2\mu_1 + c_{q2}\mu_2}{2m\mu_1 + \mu_2 m\rho} = \frac{2\mu_1 + c_{q2}\mu_2}{m(2\mu_1 + \mu_2\rho)} \end{aligned}$$

Since $\mathcal{N}$ is just the normal sample and has all copy 2, we similarly calculate $Prob(Y_{pq} = 1) = \frac{1}{m}$. We can now see how to derive the equation used by both [2] and [9].

$$\frac{E[X_q]}{E[Y_q]} = \frac{P \times Prob(X_{pq} = 1)}{P \times Prob(Y_{pq} = 1)} = \frac{Prob(X_{pq} = 1)}{Prob(Y_{pq} = 1)}$$
$$= \frac{\frac{2\mu_1 + c_{j2}\mu_2}{m(2\mu_1 + \mu_2\rho)}}{\frac{1}{m}} = \frac{2\mu_1 + c_{j2}\mu_2}{2\mu_1 + \mu_2\rho}$$

C Multinomial Maximum Likelihood Solutions - Unconstrained Problem.

In real data there are two natural constraints on the interval count matrix $\mathbf{C}$: (i) entries of $\mathbf{C}$ are bounded, and (ii) the number of columns is less than the number of rows, $n < m$. In this section we show that if either of these constraints are violated, then the Maximum Likelihood Mixture Decomposition Problem (MLMDP) has a straightforward solution. The additional constraint that a sample contains the normal genome is a simple extension, which we do not include here for clarity.

Theorem C.1. *Suppose $\mathbf{r} \in \mathbb{N}^m$ is a read depth vector. If $n \geq 1$, then we can always find $\mathbf{C} \in \mathbb{N}^{m \times n}$ and $\mu \in \mathbb{R}^n$ with $\sum_{k=1}^{n} \mu_k = 1$ and $\mu_k \geq 0$ for all $k = 1, \ldots, n$ such that $(\mathbf{C}, \mu)$ is a maximum likelihood solution for $P(\mathbf{r}|\mathbf{C}, \mu) = Mult(\mathbf{r}|\widehat{\mathbf{C}\mu})$.*

Proof. Let P_m be the space of parameters for a multinomial of size m. Using Lagrange multipliers it is straightforward to show that $Mult(\mathbf{r}|\mathbf{p})$, for $\mathbf{p} \in P_m$, is maximized for $\mathbf{p}^* = \frac{\mathbf{r}}{|\mathbf{r}|_1} = (\frac{r_1}{\sum_{j=1}^{m} r_j}, \ldots, \frac{r_m}{\sum_{j=1}^{m} r_j}) = \widehat{\mathbf{r}}$. We now just need to show that we can always construct an interval count matrix $\mathbf{C}$ and genome mixing vector μ such that $\mathbf{p}^* = \widehat{\mathbf{C}\mu}$. In particular, we define $\mu = (1, 0, 0, \ldots, 0)^T \in \mathbb{R}^n$ and $\mathbf{C} = (\mathbf{c}_1, \ldots, \mathbf{c}_n)$ where $\mathbf{c}_1 = \mathbf{r}$. We now show that $\widehat{\mathbf{C}\mu} = \mathbf{p}^*$.

$$\widehat{\mathbf{C}\mu} = \frac{\mathbf{C}\mu}{|\mathbf{C}\mu|_1} = \frac{\mathbf{r}}{|\mathbf{r}|_1} = \mathbf{p}^*$$

Therefore $(\mathbf{C}, \mu)$ is a maximum likelihood solution for $P(\mathbf{r}|\mathbf{C}, \mu) = Mult(\mathbf{r}|\widehat{\mathbf{C}\mu})$. □

Theorem C.2. *Suppose $\mathbf{r} \in \mathbb{N}^m$ is a read depth vector. If $n \geq m$, then we can always find $\mathbf{C} \in \mathbb{N}^{m \times n}$ and $\mu \in \mathbb{R}^n$ with $\sum_{k=1}^{n} \mu_k = 1$ and $\mu_k \geq 0$ for all $k = 1, \ldots, n$ such that $(\mathbf{C}, \mu)$ is a maximum likelihood solution for $P(\mathbf{r}|\mathbf{C}, \mu) = Mult(\mathbf{r}|\widehat{\mathbf{C}\mu})$.*

Proof. For a given $\mathbf{r}$ and fixed m and n with $n \geq m$ we will construct $\mathbf{C}$ and μ such that $(\mathbf{C}, \mu)$ is a maximum likelihood solution. First we define $\widehat{\mathbf{r}} = \frac{\mathbf{r}}{|\mathbf{r}|_1}$. We now explicitly choose $\mu = (\widehat{r}_1, \ldots, \widehat{r}_m, 0, \ldots, 0)$ (which we can do since $n \geq m$). By construction $\mu \in \mathbb{R}^n$, $\sum_{k=1}^{n} \mu_k = 1$ and $\mu_k \geq 0$ for all $k = 1, \ldots, n$. We now explicitly choose $\mathbf{C}$ such that $c_{ii} = 1$ for $i = 1, \ldots, m$ and 0 for all other entries. We now show that $\widehat{\mathbf{C}\mu} = \widehat{\mathbf{r}}$.

$$\widehat{\mathbf{C}\mu} = \frac{\mathbf{C}\mu}{|\mathbf{C}\mu|_1} = \frac{\widehat{\mathbf{r}}}{|\widehat{\mathbf{r}}|_1} = \widehat{\mathbf{r}}$$

□

D Proof of Theorem 1

We provide the full proof of Theorem 1 here.

Theorem 1. *Suppose* $\mathbf{p} \in P_\Omega$*, so* $\mathbf{p} = \widehat{\mathbf{C}\mu}$ *for some* $(\mathbf{C}, \mu) \in \Omega$*. Then there exists* $\mu' \in \Delta_{n-1}$ *such that* $\mathbf{p} = \widehat{\mathbf{C}}\mu'$*, where* $\widehat{\mathbf{C}} = (\widehat{\mathbf{c}_1}, \ldots, \widehat{\mathbf{c}_n})$*.*

Proof. Let $\mu' = (\mu'_1, \ldots, \mu'_n)$ where $\mu'_j = \frac{\mu_j |\mathbf{c}_j|_1}{\sum_{h=1}^n \mu_h |\mathbf{c}_h|_1}$. By definition $\sum_{j=1}^n \mu'_j = 1$ and $\mu'_j \geq 0$, so $\mu' \in \Delta_{n-1}$. We now show that $\widehat{\mathbf{C}}\mu' = \mathbf{p}$. For each $j \in \{1, \ldots, m\}$ we compute that the ith entry of $\widehat{\mathbf{C}}\mu'$ is:

$$
\begin{aligned}
(\widehat{\mathbf{C}}\mu')_i &= \sum_{j=1}^n \widehat{c}_{ij}\mu'_j = \sum_{j=1}^n \frac{c_{ij}}{|\mathbf{c}_j|_1} \frac{\mu_j |\mathbf{c}_j|_1}{\sum_{h=1}^n \mu_h |\mathbf{c}_h|_1} \\
&= \sum_{j=1}^n \frac{\mu_j c_{ij}}{\sum_{h=1}^n \mu_h |\mathbf{c}_h|_1} = \frac{\sum_{j=1}^n \mu_j c_{ij}}{\sum_{h=1}^n \sum_{g=1}^m \mu_h c_{gh}} \\
&= (\widehat{\mathbf{C}\mu})_i = p_i.
\end{aligned}
$$

Hence, we see that $\mathbf{p} = \widehat{\mathbf{C}}\mu'$. □

As described in the main text, Theorem 1 allows us to solve separate convex optimization problems in the space P_Ω. Figure S2 shows an example of the geometry of the problem when $m = 4$ and $n = 3$.

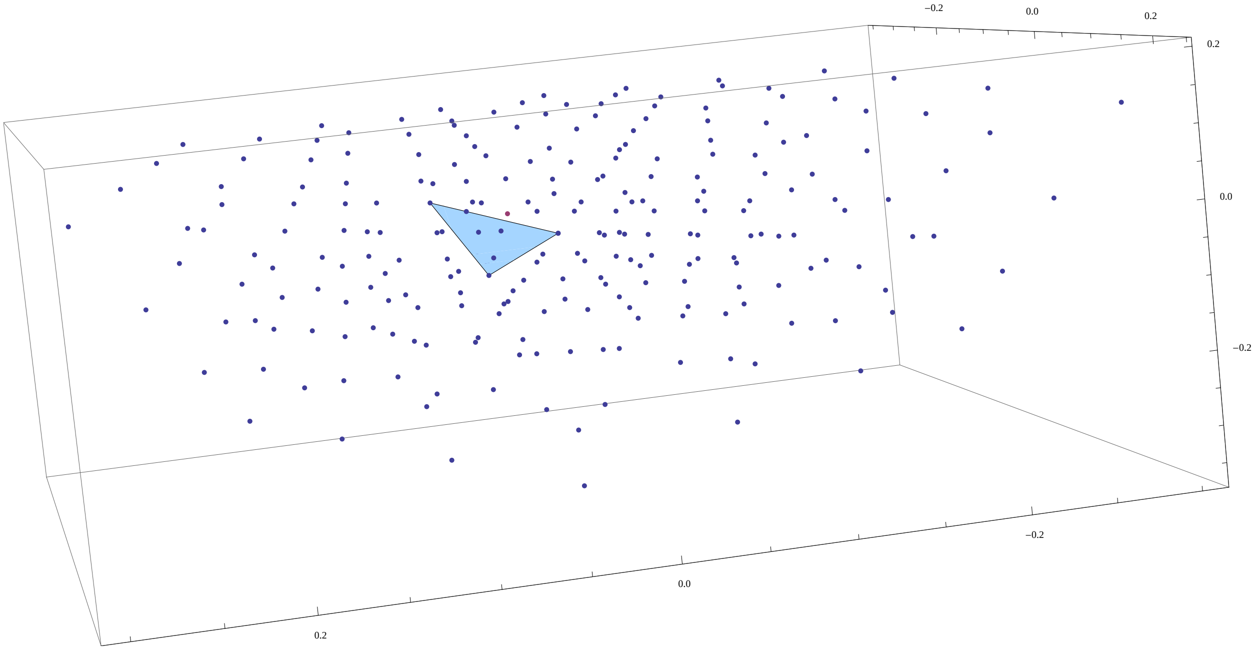

Figure S2: When $n = 3$, convex combinations of normalized columns in $\mathbf{C}$ form planes, or 2-simplices, which are embedded in Δ_{m-1} (as described in Theorem 1). Here we show an example with $m = 4$ where one such plane is highlighted in blue.

E Efficiently Restricting the search space $\mathcal{C}_{m,n,k}$

In this section, we first provide the proof of Theorem 2, which we use to show how we restrict the space $\mathcal{C}_{m,n,k}$ while looking for an optimal interval count matrix $\mathbf{C}$. We then present a polynomial (in m) algorithm for solving MLMDP problem for $n = 2$, and finally, we discuss how we can shrink the search space in the case when $n > 2$.

E.1 Proof of Theorem 2

Theorem 2. *Suppose* $\mathbf{p}^* = \widehat{\mathbf{C}\mu} = \underset{p \in P_{\Omega_{m,n,k}}}{\operatorname{argmin}} \mathcal{L}_{\mathbf{r}}(\mathbf{p})$. *Then we have the following.*

1. $\mathbf{p}^*$ *and* $\mathbf{r}$ *have compatible order.*
2. *If* $n = 2$ *and* $\mu_2^* > 0$, *then* $\mathbf{r}$ *and* $\mathbf{c}_2^*$ *have compatible order.*

Proof. We start with part (1) and proceed by contradiction. Suppose $\mathbf{r}$ and $\mathbf{p}$ do not have a compatible order, that is there exist $i, j \in \{1, \ldots, m\}$ such that $r_i > r_j$, but $p_i < p_j$. Without loss of generality assume $i < j$, and let $\mathbf{p}'$ be a point on the simplex obtained from $\mathbf{p}$ by swapping the ith and the jth entries. We first show that $\mathbf{p}' \in P_{\Omega_{m,n,k}}$. Since $\mathbf{p} \in P_{\Omega_{m,n,k}}$, by definition there exists a $(\mathbf{C}, \mu) \in \Omega_{m,n,k}$ such that $\mathbf{p} = \widehat{\mathbf{C}\mu}$. Let $\mathbf{C}'$ be a matrix obtained from $\mathbf{C}$ by swapping the ith and the jth rows. We define $\mathbf{p}' = \widehat{\mathbf{C}'\mu}$. Since the set of entries in $\mathbf{C}'$ is as same as the set of entries in $\mathbf{C}$, we have $(\mathbf{C}', \mu) \in \Omega_{m,n,k}$. Thus $\mathbf{p}' \in P_{\Omega_{m,n,k}}$.

Now, by the theorem assumption $\mathcal{L}_{\mathbf{r}}(\mathbf{p})$ is minimized, and therefore $\mathcal{L}_{\mathbf{r}}(\mathbf{p}) \leq \mathcal{L}_{\mathbf{r}}(\mathbf{p}') \Rightarrow \mathcal{L}_{\mathbf{r}}(\mathbf{p}) - \mathcal{L}_{\mathbf{r}}(\mathbf{p}') \leq 0$. We define $\delta = r_i - r_j > 0$. We have

$$
\begin{aligned}
\mathcal{L}_{\mathbf{r}}(\mathbf{p}) - \mathcal{L}_{\mathbf{r}}(\mathbf{p}') &= (-\sum_{i=1}^{m} r_i \log(p_i) + \alpha) - (-\sum_{i=1}^{m} r_i \log(p_i') + \alpha) \\
&= -r_i \log(p_i) - r_j \log(p_j) + r_i \log(p_j) + r_j \log(p_i) \\
&= -(r_j + \delta) \log(p_i) - r_j \log(p_j) + (r_j + \delta) \log(p_j) + r_j \log(p_i) \\
&= -r_j \log(p_i) - \delta \log(p_i) - r_j \log(p_j) + r_j \log(p_j) + \delta \log(p_j) + r_j \log(p_i) \\
&= -\delta \log(p_i) + \delta \log(p_j) \\
&= \delta(\log(p_j) - \log(p_i)) \\
&> 0.
\end{aligned}
$$

This is a contradiction. Therefore, it must be the case that $\mathbf{r}$ and $\mathbf{p}$ have compatible order, thus completing the proof of part (1).

Part (2) following directly form part (1) since we assume that the first column of $\mathbf{C}$, denoted $\mathbf{c}_1$ has equal entries. $\square$

E.2 An Efficient Algorithm for $n = 2$

A permutation $\pi : \{1, \ldots, m\} \to \{1, \ldots, m\}$ is $\mathbf{r}$-*compatible* if $r_{\pi_1} \leq r_{\pi_2} \leq \ldots, \leq r_{\pi_m}$. For every r-compatible permutation π, we define $M_{\mathbf{r},\pi}$ to be the set of matrices in $\mathbf{C} \in \mathcal{C}_{m,2,k}$ where $\mathbf{c}_2$ and $\mathbf{r}_{\pi(1)}, \ldots, \mathbf{r}_{\pi(m)}$ have compatible order.

By Theorem 2 we only need to enumerate the elements of sets $M_{\mathbf{r},\pi}$ for each r-compatible permutation π. For a specific π the number of elements in $M_{\mathbf{r},\pi}$ is $\binom{m+k}{k}$, and we can enumerate $M_{\mathbf{r},\pi}$ efficiently. If r_i's are all distinct (which is expected for real data) then the r-compatible permutation π is unique, and our search space will drop from $O(k^m)$ to $O\big(\binom{m+k}{k}\big) = O(m^k)$, meaning the exponential size search space reduces to a polynomial (in m) size space.

Here we show how we can efficiently enumerate all the elements in $M_{\mathbf{r},\pi}$. Suppose the matrices $\mathbf{C} = (\mathbf{c}_1, \mathbf{c}_2) \in M_{\mathbf{r},\pi}$, where $\mathbf{c}_1 = \mathbf{2}^m$ and $\mathbf{c}_2 = (c_{12}, \ldots, c_{m2})^T$, are ordered based on the lexicographical order of $(c_{\pi(1)2}, \ldots, c_{\pi(m)2})$. Given a matrix $\mathbf{C}' \in M_{\mathbf{r},\pi}$, Algorithm 1 finds the *next* matrix in $M_{\mathbf{r},\pi}$, i.e., the successor of the matrix $\mathbf{C}'$ in $M_{\mathbf{r},\pi}$, in time $O(m)$ which implies that all the matrices in $M_{r,\pi}$ can be enumerated in time $O(m^{k+1})$ since the size of $M_{r,\pi}$ is $O(m^k)$.

Algorithm 1: Enumeration of matrices in $M_{\mathbf{r},\pi}$.

input : A matrix $\mathbf{C} \in M_{\mathbf{r},\pi}$
output: The next matrix $\texttt{next}(\mathbf{C}) \in M_{\mathbf{r},\pi}$

```
begin
    (c_{1,2}, ..., c_{m,2}) ⟵ the second column of C;
    j ⟵ the largest j such that c_{π(j),2} < k;
    if j exists then
        for i = 1 → j − 1 do
            c'_{π(i),2} ⟵ c_{π(i),2};
        for i = j → m do
            c'_{π(i),2} ⟵ c_{π(j),2} + 1;
    else
        next(C) ⟵ NULL;
    next(C) ⟵ the matrix with columns 2^m and (c'_{1,2}, ..., c'_{m,2})^T;
    return next(C);
```

E.3 Restricting the solution space when $n > 2$

Now suppose $n > 2$. Based on Theorem 2 if $\mathbf{p}^* = \widehat{\mathbf{C}^*\mu^*} = \underset{p \in P_{\Omega_{m,n,k}}}{\operatorname{argmin}} \mathcal{L}_{\mathbf{r}}(\mathbf{p})$, then $\widehat{\mathbf{C}^*\mu^*}$ and $\mathbf{r}$ have compatible order. Therefore, $\forall i, j \in \{1, \ldots, m\}$ we have

$$\mathbf{r}_i \leq \mathbf{r}_j \Leftrightarrow (\widehat{\mathbf{C}^*\mu^*})_i \leq (\widehat{\mathbf{C}^*\mu^*})_j \Leftrightarrow (\mathbf{C}^*\mu^*)_i \leq (\mathbf{C}^*\mu^*)_j \Rightarrow \exists\, t \in \{1, \ldots, n\},\ c^*_{i,t} \leq c^*_{j,t}.$$

Although this does not impose any total order on columns of $\mathbf{C}$, it still puts some restrictions on entries of $\mathbf{C}$. This leads into a smaller search space with exponential size (in m and n), but however, this can be useful to speed up the heuristic algorithms.

F Separable Convexity of $\mathcal{L}_{\mathbf{r}}(\mathbf{p})$

In this section we show that our objective function is separable convex.

Lemma F.1. $\mathcal{L}_{\mathbf{r}}(\mathbf{p}) = -\sum_{j=1}^{m} r_j \log(p_j) + \alpha$ *is separable convex for* $\mathbf{p} \in P_m$.

Proof. To show that $\mathcal{L}_{\mathbf{r}}(\mathbf{p})$ is a separable convex function for $\mathbf{p} \in P_m$, we show (i) P_m is a convex space; (2) the functions $\ell_j(p_j) = -r_j \log(p_j)$ are convex. Finally since $\mathcal{L}_{\mathbf{r}}(\mathbf{p}) = \sum_{j=1}^{m} \ell_j(p_j) + \alpha$ we conclude the separable convexity of $\mathcal{L}_{\mathbf{r}}(\mathbf{p})$.

Suppose $\lambda \in [0, 1]$ and $\mathbf{p}, \mathbf{q} \in P_m$ are arbitrarily chosen. Let $\mathbf{s} = \lambda\mathbf{p} + (1 - \lambda)\mathbf{q}$. By definition $s_j = \lambda p_j + (1 - \lambda)q_j \geq 0$ since $p_j, q_j, \lambda \geq 0$, and $\sum_{j=1}^{m} s_j = 1$ since

$$\sum_{j=1}^{m} s_j = \lambda \sum_{j=1}^{m} p_j + (1 - \lambda) \sum_{j=1}^{m} q_j = \lambda + (1 - \lambda) = 1 \Rightarrow \mathbf{s} \in P_m, \text{ and } P_m \text{ is convex.}$$

Now, for any $j \in \{1, \ldots, m\}$

$$\begin{aligned} \ell_j(s_j) = \ell_j(\lambda p_j + (1 - \lambda)q_j) &= -r_j \log(\lambda p_j + (1 - \lambda)q_j) \\ &\leq -r_j(\lambda \log(p_j) + (1 - \lambda) \log(q_j)) && \text{(By Jensen's Inequality)} \\ &= -r_j\lambda \log(p_j) - r_j(1 - \lambda) \log(q_j) \\ &= \lambda\ell_j(p_j) + (1 - \lambda)\ell_j(q_j) \\ &\Rightarrow \text{the function } \ell_j(p_j) \text{ is a convex function.} \end{aligned}$$

Finally, since α is a constant and $\mathcal{L}_{\mathbf{r}}(\mathbf{p}) = \sum_{j=1}^{m} \ell_j(p_j) + \alpha$, the function $\mathcal{L}_{\mathbf{r}}(\mathbf{p})$ is separable convex.

□

G Proof of the Theorem 3

In this section we present the proof for Theorem 3. To do so, we first prove two important properties of the function Φ, and next we show there is a unique $\mathbf{p} \in \Phi(\mathbf{C}\mu)$, where $(\mathbf{C}, \mu) \in \Omega_{m,n}$, such that $\mathcal{L}_{\mathbf{r}}(\mathbf{p})$ is minimized.

Lemma G.1. *The function* Φ *has the following properties:*

(i) $\Phi(\mathbf{v}) = \Phi(\widehat{\mathbf{v}})$*, for all* $\mathbf{v} \in \mathbb{R}^m$*, and*

(ii) restrictions of Φ *and* Φ^{-1} *to* Δ_{m-1} *are inverse of each other.*

Proof. First we show that $\Phi(\mathbf{v}) = \Phi(\widehat{\mathbf{v}})$:

$$\Phi(\mathbf{v}) = \widehat{\mathbf{W}\mathbf{v}} = \frac{\mathbf{W}\mathbf{v}}{|\mathbf{W}\mathbf{v}|_1} = \frac{\frac{\mathbf{W}\mathbf{v}}{|\mathbf{v}|_1}}{\frac{|\mathbf{W}\mathbf{v}|_1}{|\mathbf{v}|_1}} = \frac{\mathbf{W}\frac{\mathbf{v}}{|\mathbf{v}|_1}}{|\mathbf{W}\frac{\mathbf{v}}{|\mathbf{v}|_1}|_1} = \frac{\mathbf{W}\widehat{\mathbf{v}}}{|\mathbf{W}\widehat{\mathbf{v}}|_1} = \Phi(\widehat{\mathbf{v}}). \tag{1}$$

By definition of Φ^{-1} we have $\Phi^{-1}(\mathbf{q}) = \widehat{\mathbf{W}^{-1}\mathbf{q}}$. Let $\mathbf{p} \in \Delta_{m-1}$:

$$\Phi^{-1}(\Phi(\mathbf{p})) = \Phi^{-1}(\widehat{\mathbf{Wp}}) = \Phi^{-1}(\frac{\mathbf{Wp}}{|\mathbf{Wp}|_1}) = (\widehat{\mathbf{W}^{-1}\frac{\mathbf{Wp}}{|\mathbf{Wp}|_1}}) = \frac{\mathbf{W}^{-1}\frac{\mathbf{Wp}}{|\mathbf{Wp}|_1}}{|(\mathbf{W}^{-1}\frac{\mathbf{Wp}}{|\mathbf{Wp}|_1})|_1}$$

$$= \frac{\frac{\mathbf{p}}{|\mathbf{Wp}|_1}}{\frac{|\mathbf{p}|_1}{|\mathbf{Wp}|_1}} = \frac{\mathbf{p}}{|\mathbf{p}|_1} = \mathbf{p},$$

where the last equation comes from the fact that $\mathbf{p} \in \Delta_{m-1}$ and $|\mathbf{p}|_1 = 1$. Using the same argument $\Phi(\Phi^{-1}(\mathbf{p})) = \mathbf{p}$, and the proof is complete. □

Theorem G.1. $\left| \underset{\mathbf{p}=\Phi(\mathbf{C}\mu),(\mathbf{C},\mu)\in\Omega_{m,n}}{\operatorname{argmin}} \mathcal{L}_{\mathbf{r}}(\mathbf{p}) \right| = 1.$

Proof. Let $\mu^* = (1, 0, 0, \dots, 0)^T \in \mathbb{R}^n$ and $\mathbf{C}^*$ be a matrix whose first column is $(c_1^*, \dots, c_m^*)^T$, where $c_i^* = (\prod_j w_j) \cdot \frac{r_i}{w_i} \in \mathbb{Z}$, and other entries of $\mathbf{C}$ are arbitrary integers in $\mathbb{N}$. Define $\mathbf{p}^* = \Phi(\mathbf{C}^*\mu^*)$. We claim that $\mathbf{p}^*$ is the only element in the set $\underset{\mathbf{p}=\Phi(\mathbf{C}\mu),(\mathbf{C},\mu)\in\Omega_{m,n}}{\operatorname{argmin}} \mathcal{L}_{\mathbf{r}}(\mathbf{p})$.

First, $\mathbf{p}^* \in \underset{\mathbf{p}=\Phi(\mathbf{C}\mu),(\mathbf{C},\mu)\in\Omega_{m,n}}{\operatorname{argmin}} \mathcal{L}_{\mathbf{r}}(\mathbf{p})$: by definition we have

$$p_i^* = \Phi(\mathbf{C}^*\mu^*)_i = \frac{c_i^* \cdot w_i}{\sum_j c_j^* \cdot w_j} = \frac{(\prod_j w_j)\frac{r_i}{w_i} \cdot w_i}{\sum_h \frac{r_h \prod_j w_j}{w_h} \cdot w_h} = \frac{(\prod_j w_j) r_i}{(\prod_j w_j) \sum_h r_h} = \frac{r_i}{\sum_h r_h} = (\widehat{\mathbf{r}})_i.$$

This implies that $\mathbf{p}^* \in \underset{\mathbf{p}\in\Delta_{m-1}}{\operatorname{argmin}} \mathcal{L}_{\mathbf{r}}(\mathbf{p})$, and since $\{\Phi(\mathbf{C}\mu) \mid (\mathbf{C}, \mu) \in \Omega_{m,n})\} \subset \Delta_{m-1}$ we have $\mathbf{p}^* \in \underset{\mathbf{p}=\Phi(\mathbf{C}\mu),(\mathbf{C},\mu)\in\Omega_{m,n}}{\operatorname{argmin}} \mathcal{L}_{\mathbf{r}}(\mathbf{p})$.

Now suppose $\mathbf{p}' \in \underset{\mathbf{p}=\Phi(\mathbf{C}\mu),(\mathbf{C},\mu)\in\Omega_{m,n}}{\operatorname{argmin}} \mathcal{L}_{\mathbf{r}}(\mathbf{p})$. Thus, $\mathcal{L}_{\mathbf{r}}(\mathbf{p}') = \mathcal{L}_{\mathbf{r}}(\mathbf{p}^*)$, and $\mathbf{p}' \in \underset{\mathbf{p}\in\Delta_{m-1}}{\operatorname{argmin}} \mathcal{L}_{\mathbf{r}}(\mathbf{p})$, as we showed $\mathbf{p}^* \in \underset{\mathbf{p}\in\Delta_{m-1}}{\operatorname{argmin}} \mathcal{L}_{\mathbf{r}}(\mathbf{p})$. But $\mathbf{p}' = \mathbf{p}^*$, since there is a unique optimal point for $\underset{\mathbf{p}\in\Delta_{m-1}}{\operatorname{argmin}} \mathcal{L}_{\mathbf{r}}(\mathbf{p})$, i.e., $|\underset{\mathbf{p}\in\Delta_{m-1}}{\operatorname{argmin}} \mathcal{L}_{\mathbf{r}}(\Phi(\mathbf{p}))| = 1$, which completes the proof for uniqueness of $\mathbf{p}^*$. □

Theorem 3. *Let $\Phi^{-1} : \mathbb{R}^m \to \mathbb{R}^m$ be $\Phi^{-1}(\mathbf{v}) = \widehat{W^{-1}\mathbf{v}}$. We have the following set equality,*

$$\underset{(\mathbf{C},\mu)\in\Omega_{m,n}}{\operatorname{argmin}} \mathcal{L}_{\mathbf{r}}(\Phi(\mathbf{C}\mu)) = \underset{(\mathbf{C},\mu)\in\Omega_{m,n}}{\operatorname{argmin}} \mathcal{L}_{\Phi^{-1}(\mathbf{r})}(\widehat{\mathbf{C}\mu}).$$

Proof. By Theorem G.1 $\underset{\mathbf{p}=\mathbf{C}\mu,(\mathbf{C},\mu)\in\Omega_{m,n}}{\operatorname{argmin}} \mathcal{L}_{\mathbf{r}}(\mathbf{p})$ has a unique point $\mathbf{p}^*$, where $p_i^* = \frac{r_i}{\sum_{j=1}^m r_j}$. There is a unique optimal point $\mathbf{q}^*$ in $\underset{(\mathbf{C},\mu)\in\Omega_{m,n}}{\operatorname{argmin}} \mathcal{L}_{\Phi^{-1}(\mathbf{r})}(\widehat{\mathbf{C}\mu})$, where $q_i^* = \frac{\frac{r_i}{w_i}}{\sum_{j=1}^m \frac{r_j}{w_j}}$. Now we have

$$\begin{aligned}
(\mathbf{C}^*, \mu^*) \in \underset{(\mathbf{C},\mu)\in\Omega_{m,n}}{\operatorname{argmin}}\ \mathcal{L}_{\mathbf{r}}(\Phi(\mathbf{C}\mu)) &\Leftrightarrow \mathcal{L}_{\mathbf{r}}(\Phi(\mathbf{C}^*\mu^*) = \mathcal{L}_{\mathbf{r}}(\mathbf{p}^*) \\
\text{(using uniqueness of } \mathbf{p}^*\text{, and Lemma G.1)} &\Leftrightarrow \Phi(\widehat{\mathbf{C}^*\mu^*}) = \Phi(\mathbf{C}^*\mu^*) = \mathbf{p}^* = \frac{\mathbf{r}}{\sum_j r_j} = \widehat{\mathbf{r}} \\
\text{(applying } \Phi^{-1} \text{ and using Lemma G.1)} &\Leftrightarrow \widehat{\mathbf{C}^*\mu^*} = \Phi^{-1}(\widehat{\mathbf{r}}) \\
\text{(by Lemma G.1)} &\Leftrightarrow \widehat{\mathbf{C}^*\mu^*} = \Phi^{-1}(\mathbf{r}) \\
\text{(since } \Phi^{-1}(\mathbf{r}) \in \Delta_{m-1}, |\Phi^{-1}(\mathbf{r})|_1 = 1) &\Leftrightarrow \widehat{\mathbf{C}^*\mu^*} = \frac{\Phi^{-1}(\mathbf{r})}{|\Phi^{-1}(\mathbf{r})|_1} = \left(\frac{\frac{r_1}{w_1}}{\sum_{j=1}^m \frac{r_j}{w_j}}, \dots, \frac{\frac{r_m}{w_m}}{\sum_{j=1}^m \frac{r_j}{w_j}}\right) \\
&\Leftrightarrow \mathcal{L}_{\Phi^{-1}(\mathbf{r})}(\widehat{\mathbf{C}^*\mu^*}) = \mathcal{L}_{\Phi^{-1}(\mathbf{r})}(\mathbf{q}^*) \\
&\Leftrightarrow (\mathbf{C}^*, \mu^*) \in \underset{(\mathbf{C},\mu)\in\Omega_{m,n}}{\operatorname{argmin}}\ \mathcal{L}_{\Phi^{-1}(\mathbf{r})}(\widehat{\mathbf{C}\mu}).
\end{aligned}$$

□

H Compatible Order in the Weighted Case

As discussed in the main paper, while we do not expect that $\operatorname{argmin}_{(\mathbf{C},\mu)\in\Omega_{m,2,k}} \mathcal{L}_{\mathbf{r}}(\Phi(\mathbf{C}\mu))$ is equal to $\operatorname{argmin}_{(\mathbf{C},\mu)\in\Omega_{m,2,k}} \mathcal{L}_{\Phi^{-1}(\mathbf{r})}(\widehat{\mathbf{C}\mu})$, we may reasonably assume that because the unconstrained optimum are equal, the transformation Φ is linear, and the objective function is well-behaved (separable convex), that a solution to $\operatorname{argmin}_{(\mathbf{C},\mu)\in\Omega_{m,2,k}} \mathcal{L}_{\mathbf{r}}(\Phi(\mathbf{C}\mu))$ will satisfy the name order constraints as $\mathcal{L}_{\Phi^{-1}(\mathbf{r})}(\widehat{\mathbf{C}\mu})$. Namely, we expect that the solution will have compatible order with $\Phi^{-1}(\mathbf{r})$ (Theorem 2). We verified this conjecture with simulations similar to those in the main paper with the change that both $\mathbf{r}$ and $\mathbf{w}$ were chosen at random. We then verified that the solution set returned when all matrices $\mathbf{C} \in \mathcal{C}_{m,n,k}$ were considered, was the same as when only $\mathbf{C}$ with compatible order to $\Phi^{-1}(\mathbf{r})$ were considered. We ran for values of k from 1 up to 7, and always obtained the same solution set.

I Data Simulation

In this section we describe our methods for data simulation of sequencing data, corresponding SNP array data, and synthetic mixtures of real sequencing data.

Simulated Sequencing Data with $n = 2$ We randomly simulate sequencing data using for a mixture of tumor and normal cells ($n = 2$) using the following the following procedure. For the interval partition $\mathbf{I}$ we use the 39 autosomal chromosome arms excluding the 5 acro-centric p-arms on chromosomes 13,14,15,21, and 22. We sample an interval count matrix $\mathbf{C}$ uniformly at random from $\mathcal{C}_{39,2,k}$. For each simulated tumor sample, we draw a value μ_2 uniformly from the interval $[0.5, 0.95]$, values of tumor fraction that are reasonable for real cancer sequencing data. The expected distribution of reads is then $\mathbf{p} = \Phi(\mathbf{C}\mu)$, where the weight vector ω, is obtained from paired end sequencing data from 9 normal human genomes from [5]. ω is determined by first counting the number of concordant read pairs (with mapping quality ≥ 30) that align to the interval partition $\mathbf{I}$ for all 9 genomes. We then average the observed distribution of reads over these intervals to obtain a mean observed weight vector ω which we then normalize to obtain a valid multinomial

parameter.

Under perfect conditions, the read depth vector $\mathbf{r}$ and weight vector $\mathbf{w}$ are drawn directly from the multinomial distributions with parameters $\mathbf{p}$ and ω. We simulate errors in the sequencing and analysis process by adding noise to $\mathbf{r}$ and $\mathbf{w}$. These errors occur for a variety of reasons. First, copy number aberrations that change the length of the cancer genome, but are not appropriately represented in $\mathbf{I}$, may *globally* alter the observed read depth over all intervals. We model this noise by drawing vectors $\mathbf{r}_0$ and $\mathbf{w}_0$ from a Dirichlet prior with parameters proportional to $\mathbf{p}$ and ω such that the expected number of reads corresponds to 30X coverage of the normal genome. Second, additional sources of noise in read depth estimation occur due to sequencing errors or alignment errors caused by repetitive regions. To model these errors, we add Gaussian noise to each entry of r_i of $\mathbf{r}_0$ (resp. w_i of $\mathbf{w}_0$) using a Gaussian distribution with mean 0 and standard deviation ϕr_i (resp. ϕw_j).

Simulated Sequencing Data with $n = 3$ We construct simulated sequencing data for a mixture of normal cell and two cancer subpopulations ($n = 3$ genomes in the mixture) using the same procedure as for $n = 2$ genomes with the following changes: (1) We require the content of each tumor component to be greater than 20%; (2) our set of intervals is just the first m q-arms and (3) we randomly sample $\mathbf{C}$ such that it contains a fixed number of amplifications and a random number of heterozygous deletions (similar to the real genomes we analyze). We set individual lower and upper bounds on the copy number for each interval using the same heuristic we use for real data (Supplemental Material Section N).

SNP Array To compare our algorithm for sequencing data to the algorithm ASCAT [9], designed for SNP data, we devised a method for simulating sequencing data and then converting that data to SNP array data in the format required by ASCAT. We initially create read depth data using the process described in the previous section. For both the tumor and normal genome we then create LogR and B allele-frequencies (BAF) values (necessary values for running ASCAT) for the 907,693 SNP positions on the 22 autosomes queried by the Affymetrix 6.0 SNP array. The LogR value for the tumor sample is $\log_2$ of the ratio of the tumor to normal read counts for the interval containing the SNP location. The LogR ratio for the normal sample is 0, indicating a copy number of 2. We randomly determine A_N and B_N, the number of A and B alleles for each SNP in the normal genome. We then draw a total read count for the SNP from a poisson distribution with parameter equal to the observed coverage for the genomic interval containing the SNP. We then simulate the number reads with the variant allele by making draws from a binomial distribution with parameter equal to the expected variant allele fraction $\frac{B_N}{A_N+B_N}$. The number of reads with the variant allele divided by the total number of reads gives the observed BAF. BAFs for the tumor sample are created in a similar manner after randomly determining the number of copies of each parental chromosome for each interval in the tumor cells. If the total number of copies for an interval is ≥ 2 we require that at least one copy of each parental chromosome is retained in the tumor cells (otherwise this implies the more complicated situation where multiple events would have occurred to the same interval). Using the data from the matched normal, we calculate A_T and B_T the number A and B alleles for each SNP in the tumor genome. We define μ_N to be the fraction of normal cells in the sample, and μ_T to be the fraction of tumor cells in the sample ($\mu_N + \mu_T = 1$). The expected variant allele fraction in the tumor sample is calculated using the following equation:

$$
\begin{aligned}
BAF =& (\frac{B_N}{A_N+B_N})\frac{\mu_N(A_N+B_N)}{\mu_N(A_N+B_N)+\mu_T(A_T+B_T)}+ \\
& (\frac{B_T}{A_T+B_T})\frac{\mu_T(A_T+B_T)}{\mu_N(A_N+B_N)+\mu_T(A_T+B_T)}
\end{aligned} \tag{2}
$$

Lastly, by default ASCAT assumes that LogR values have been scaled by a platform dependent parameter γ. We multiply all LogR values by $\gamma = 0.55$, which [9] reports as default for Illumina. We also ran all experiments with $\gamma = 1$ and find that ASCAT performs much better when $\gamma = 0.55$ and therefore present only those results.

Simulated Mixtures of Real Data In this section we describe how we created simulated mixtures of tumor cells with normal cell admixture by using real sequencing data from a matched tumor and normal AML samples. We first obtained BAM files for sample TCGA-AB-2965 obtained from CG-Hub [4]. We choose this sample because it is estimated to have 95% purity and zero copy number aberrations (as determined using array data) [4] which allows us to create datasets with realistic sequencing noise by spiking in copy number variants and mixing reads in different proportions from the tumor and normals.

We first identify all concordant read pairs where each read has mapping quality ≥ 30 and use this data to create simulated mixtures. We spike in 10 copy number variants of a fixed length (for the reported simulations we used variants of length 2.5Mb) at random non-overlapping positions in Chr20 (excluding the centromere) by up/down sampling concordant pairs. The copy number of the variant is uniformly at randomly determined to be either a deletion (heterozygous or homozygous) or an amplification (up to copy 5). We then create mixtures of this tumor genome and the matched normal by mixing together concordant pairs sampled from each. Concordant pairs are sampled with probability such that the expected total number of reads sampled from the tumor and normal samples respectively reflects a uniform sampling from all DNA in the sample given the mixing percentages and lengths of the tumor and normal genomes (while maintaining the original coverage of the original tumor sample). We then create samples of different coverage by randomly up/down sampling from this mixture. Interval partitions for Chr20 is then determined by running BIC-Seq with parameter $\lambda = 10$. We run with a smaller value of λ than we use on real data since we are only considering a single chromosome and can therefore allow for a finer partition of the reference genome into intervals. The read depth vectors $\mathbf{r}$ and $\mathbf{w}$ used as input to THetA and CNAnorm is just the count of the number of concordant pairs aligning within the derived intervals from the mixed sample and matched normal. The input to ABSOLUTE is the $\log_2$ ratio of these counts.

J Read Depth Estimation Error ϕ

In simulation we use an empirically observed value of the read depth estimation error ϕ by using the distribution of read depth, after normalizing for coverage, over the non-acrocentric chromosome arms of 9 normal samples from [5]. Over all intervals we find a mean value of $\phi = 0.037$ and median of $\phi = 0.029$. When we exclude the genome with the lowest actual coverage we observe a mean value of $\phi = 0.021$ and median of $\phi = 0.013$ (see Fig. S3).

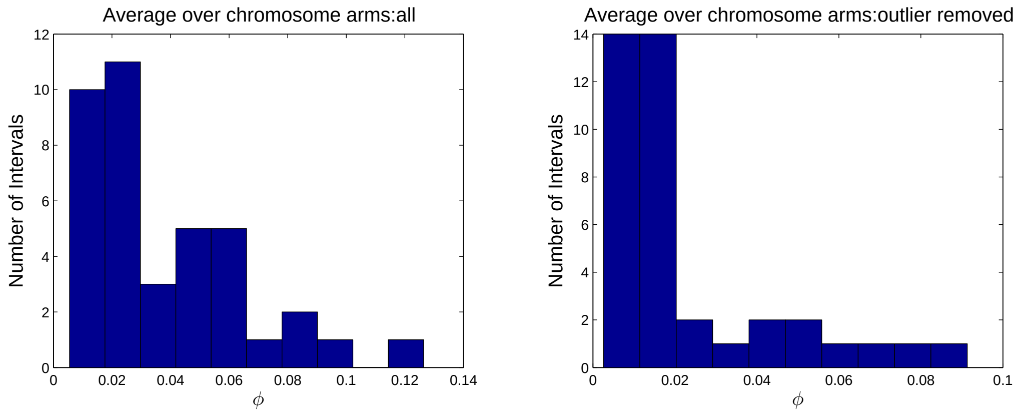

Figure S3: The distribution of the average observed ϕ over the non-acrocentric chromosome arms for 9 normal genomes (left). This distribution has mean 0.037 and median 0.029. The distribution of the average observed ϕ over the non-acrocentric chromosome arms for 8 normal genomes (right) after removal of the genome with the lowest coverage. This distribution has mean 0.021 and median 0.013.

K CNAnorm Details

Since CNAnorm first determines the set of copy numbers expressed in a sample, before estimating the sample purity, errors in the first step may result in estimated purity values above 100% (as discussed in [3]). Therefore, in simulation we only considered trials where purity was inferred $\leq 100\%$. Additionally, since CNAnorm returns non-integer copy numbers, we round their results to the nearest integer value when comparing the interval count matrices $\mathbf{C}$.

L ABSOLUTE Details

ABSOLUTE does not return a single solution, but instead returns three sets of solutions: (1) Solutions based on somatic copy number aberrations (SCNA); (2) Solutions based on recurrent Karyotypes; and (3) Solutions based on combined SCNAs and Karyotypes. Since our simulations are based on randomly generated cancer genomes, we select the ABSOLUTE solution with the highest likelihood from the SCNA category as the returned solution to compare against.

When running ABSOLUTE on simulated data, we set the maximum possible ploidy to the maximum possible value of copy number aberrations in the simulated data rather than the default value of 10. In all cases this limits the set of possible solutions considered by ABSOLUTE. All other parameters are set to their default values as described in the ABSOLUTE documentation.

M Additional Simulation Results

Here we present a more detailed analysis comparing the results of THetA, CNAnorm [3], ASCAT [9] and ABSOLUTE [2] on one of the trials from the first set of simulations in the main text. Figure S4 shows the true interval count matrix $\mathbf{C}$ and genome mixing vector μ and the inferred values by the above algorithms. The figure also includes calculations of the Copy number error and Purity error for these solutions (as defined in the main text). THetA has the most accurate purity estimate of all algorithms - being with 0.5% of the true purity and only misestimate the copy of three segments (each where the copy number estimate is

off from the true value) whereas CNAnorm, ASCAT and ABSOLUTE misestimate $17, 4$ and 37 segments respectively (out of 39 possible). The large number of copy number estimates by ABSOLUTE show the dependence between copy number estimates and purity estimates as ABSOLUTE gravely underestimates the purity of this sample.

We also ran additional experiments comparing our algorithm to CNAnorm for varying read depth estimation error ϕ, a parameter that is not relevant for the SNP array data used by ASCAT. We compare to three versions of CNAnorm [3]: (1) run with default parameters (CNAnorm), (2) run with an optional smoothing step (CNAnorm-S), and (3) run where each chromosome arm was broken into 100 intervals (CNAnorm-M) with equal read depth in each interval. In all cases, our algorithm consistently outperforms CNAnorm by a large margin – beating CNAnorm by up to 50 percentage points at estimating $\mathbf{C}$ correctly (Figure S5). Fig S6 shows the complete results for our method and CNAnorm when we analyze how well each estimates sample purity.

We also include additional simulation results for the experiments using real sequencing data to create varying mixtures of tumor and normal admixture. These results are for the simulated data presented in the main manuscript, but we provide here results where a true positive for predicting a copy number variant only requires a 50% reciprocal overlap with a true variant and a non-normal copy number predicted without requiring that the copy number be exactly correct. We find that the results for THetA change only slightly - indicating that when THetA predicts a copy number variant, it often predicts the true copy number accurately. Whereas we see significant changes in the accuracy of both CNAnorm and ABSOLUTE - indicating that these algorithms, especially for low sample purity, incorrectly estimate the overall ploidy of the tumor genomes in the mixture.

N Additional Data Processing for THetA

We assume that most of the tumor genome does not undergo focal copy number aberrations. Thus, the mode of the read depth vector provides a normal "baseline" and allows us to set tighter lower and upper bounds on the copy number for each interval. This allows us to use the following heuristics when analyzing real sequencing data.

N.1 $n = 2$

We introduce an optional heuristic that uses the distribution of $\frac{\widehat{r_j}}{\widehat{w_j}}$ over all intervals I_j to partition intervals into two groups: (1) intervals that are either deleted or have an unchanged copy number; and (2) intervals that are potentially amplified. We set different lower and upper bounds depending on which group an interval falls into.

For real sequencing data we initially make the assumption that most of the tumor genome will retain the normal expected copy number. This assumption allows us to further restrict the set of copy number values we consider for each interval. Rather than using an individual global maximum copy number value k for all intervals, we set individual lower and upper bounds for each interval using the following process. Let $\mathbf{r}$ and $\mathbf{w}$ be the observed read depth vectors for the tumor sample and matched normal samples respectively. For each interval I_j we calculate the ratio $\frac{\widehat{r_j}}{\widehat{w_j}}$, and then determine the mean x and standard deviation y for all of these ratios. For all intervals I_j where $\frac{\widehat{r_j}}{\widehat{w_j}} \leq x + by$ we set the lower bound to 0 and the upper bound to the

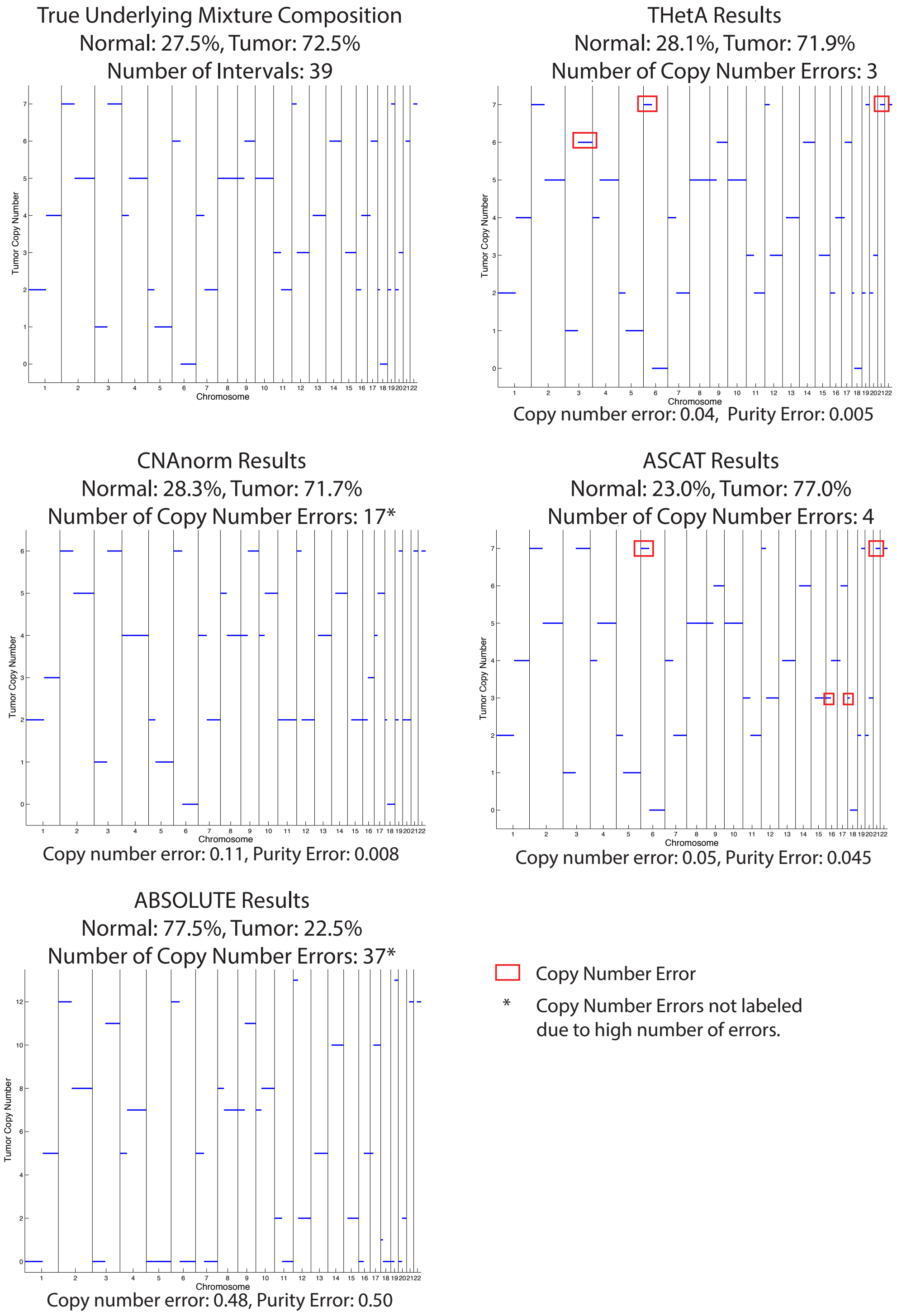

Figure S4: The true underlying interval count matrix $\mathbf{C}$ and genome mixing vector μ for one simulation along with sample reconstructions by THetA, CNAnorm, ASCAT and ABSOLUTE. Copy number error is $\frac{1}{m(n-1)}|\mathbf{C} - \mathbf{C}^*|_2$, that is, the average error per copy number estimate made, or per entry in $\mathbf{C}$, where error is the euclidean distance between $\mathbf{C}$ and $\mathbf{C}^*$. Purity error is $|\mu_2 - \mu_2^*|$, that is the distance between the true and inferred sample purity.

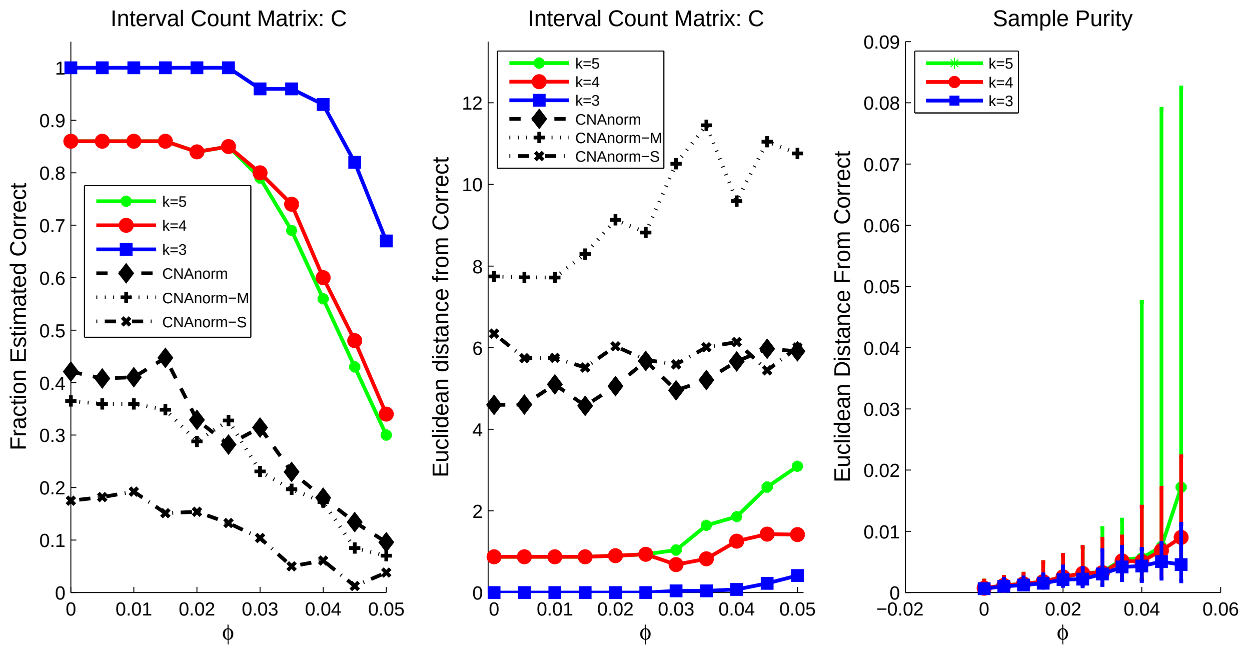

Figure S5: Comparison of our THetA algorithm and CNAnorm on simulated tumor samples with normal admixture between 5% and 50% and true underlying interval count matrix $\mathbf{C}$ sampled uniformly at random from $\mathcal{C}_{39,2,3}$. We perform 100 random trials (between 57 and 86 of which CNAnorm returns purity $< 100\%$) across varying read depth estimation error (ϕ). We also vary the maximum copy number (k) considered by THetA (beyond the true maximum copy number in the sample). We outperform CNAnorm in the metrics: (left) average number of trials where the inferred interval count matrix $\mathbf{C}$ exactly matches the true underlying interval count matrix for the sample ; (middle) average euclidean distance of between the inferred and true integer count vector $\mathbf{c}_2$ of the tumor genome; (right) median of the euclidean distance between the inferred and true genome mixing vector μ. Error bars represent the 25 and 75 percentiles. In the right figure, values for CNAnorm are not shown as the corresponding median values are between 0.07 and 0.13 – outside the range of the plot when error bars are included. (Supplemental Figure S6 gives all CNAnorm results).

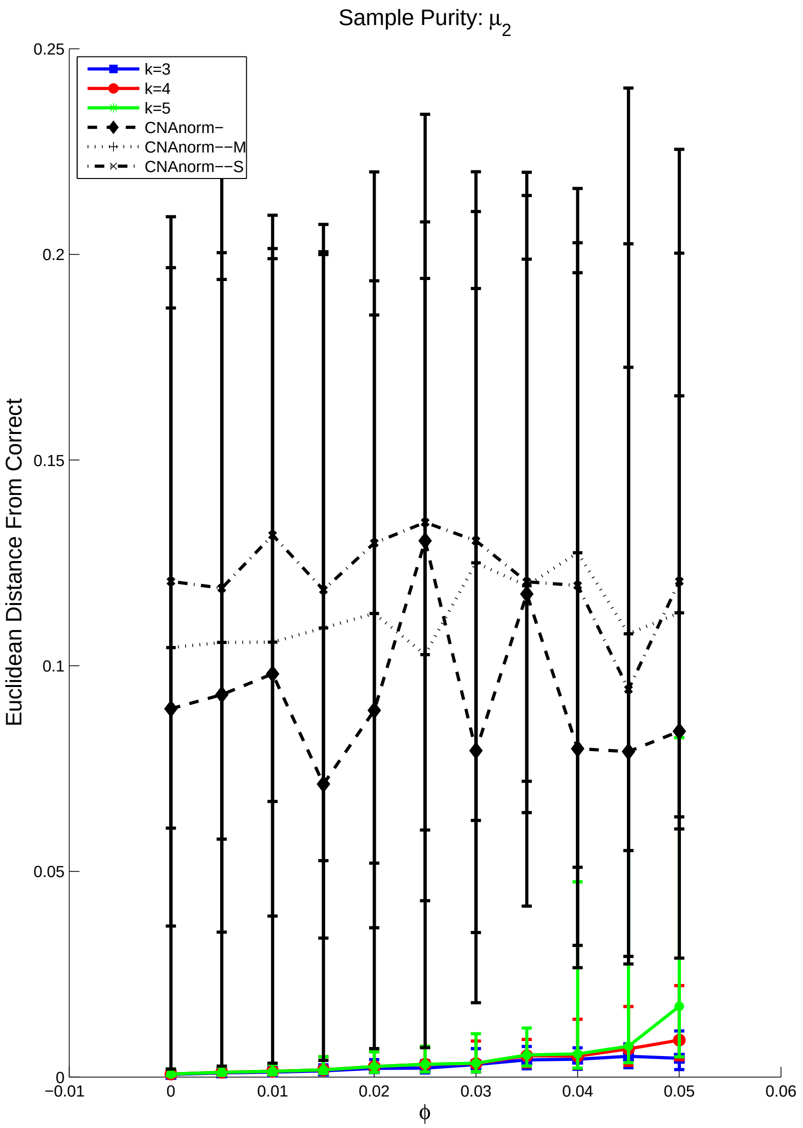

Figure S6: Effect of read depth estimation errors on a simulated data where the underlying pair $(\mathbf{C}, \mu) \sim \Omega_{39,2,3}$. ϕ is a scaling factor for the variance of Gaussian noise added to each interval. We show results for our algorithm when run with $k = 3, 4, 5$ and for three variations of the CNAnorm algorithm. We show the median of the euclidean distance of the estimated sample purity from the true underlying sample purity (where error bars represent the 25 and 75 percentiles).

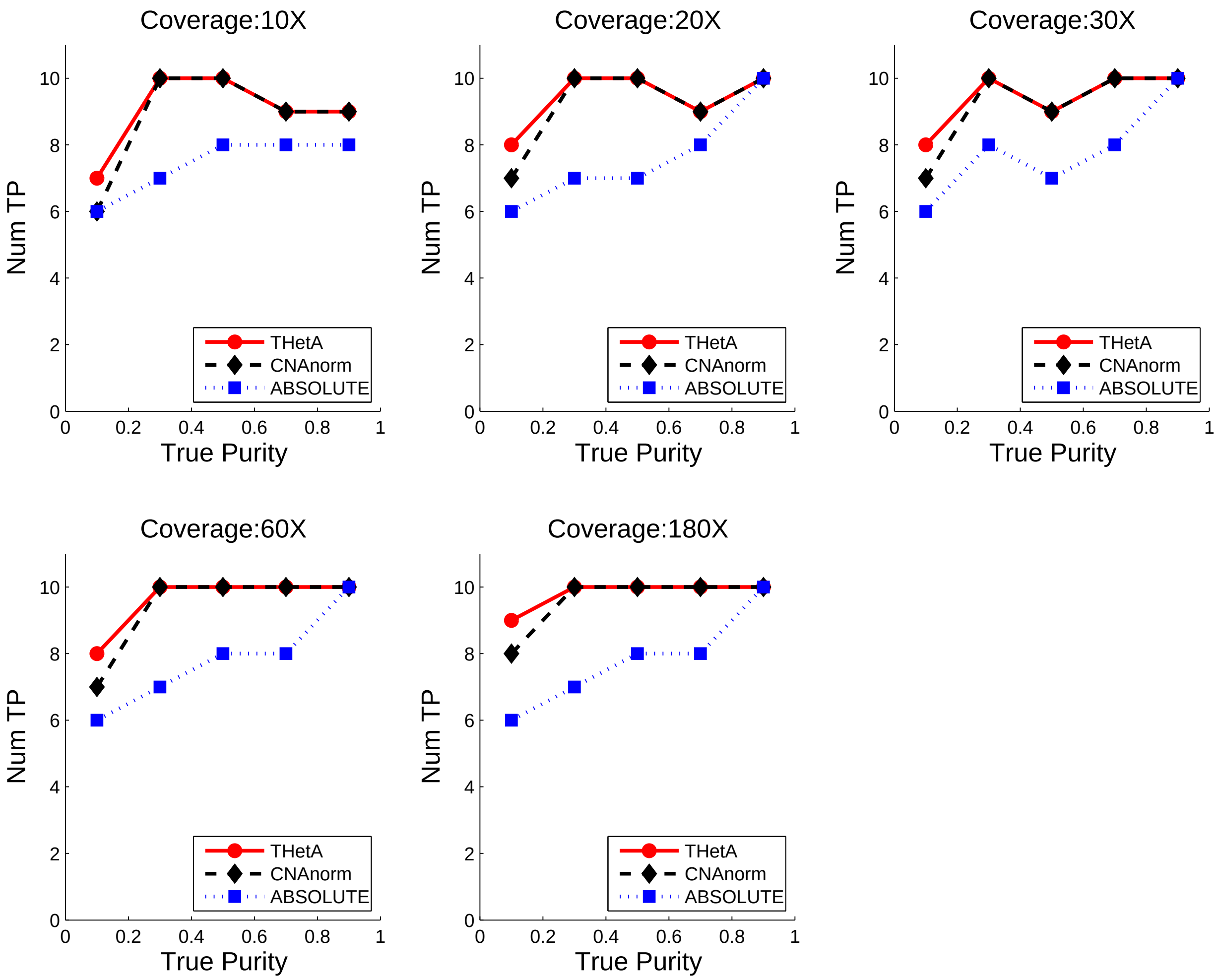

Figure S7: Comparison of true sample purity with the number of true copy number aberrations predicted (using 50% reciprocal overlap) where the copy number predicted is non-normal for THetA (T), CNAnorm (C) and ABSOLUTE (A).

expected normal copy number τ, where $b \in \mathbb{R}^+$ is a user supplied positive value. For all intervals I_j where $\frac{\widehat{r_j}}{\widehat{w_j}} > x + by$ we set the lower bound to $\max(\tau, \lceil \frac{\widehat{r_j}}{\widehat{w_j}} \rceil - 1)$ and the upper bound to $\max(k, \lceil \frac{\widehat{r_j}}{\widehat{w_j}} \rceil + 1)$. Setting b too small increases the the size of the search space, and setting b too large may lead to true amplification being missed. In practice we find that $b = 0.5$ provides a reasonable balance between these tradeoffs in many cases. However, the best value for b does change some depending on the number of copy number variants included in a sample.

In cases where most of the genome does not retain the normal expected copy number, THetA allows for the bounds set using the above heuristic to be rescaled to reflect the expected average ploidy of the sample. Additionally, since THetA returns the set of all maximum likelihood solutions, this set of solutions can contain different sample reconstructions representing different ploidys.

N.2 $n = 3$

We use a heuristic in the $n = 3$ to set bounds on the copy number for each interval that is similar to the $n = 2$ heuristic described above. For each interval in the subset of intervals that are considering for $n = 3$ we use the same lower and upper bounds on copy number estimated using the heuristic for the $n = 2$ case, with the exception that for any interval that previously had a lower bound of 0, we set a new lower bound of 1. This is required to limit the size of the search space, and is a reasonable assumption when none of the intervals in the subset being considered were predicted to be homozygously deleted by the $n = 2$ algorithm. In simulations we also set an upper bound of $k = 3$ as the genomes do not contain high-level amplifications.

N.3 Other

Since we expect tumor samples to be relatively pure we add an additional constraint to our model where we require the inferred purity of the sample to be greater than 50%.

O Read Depth Ratio Correction

One of the means we employ to validate our predictions is to use corrected read depth ratios in 50 Kb intervals over entire genomes or specific genomic intervals. We describe here the three step process for creating these corrected ratios. The first step is to normalize the read depth information for the paired tumor and normal samples to contain the same number of reads. These normalized counts are used to create initial ratios (tumor/normal). The second step is to center this distribution. For this step we select chromosomes or intervals that are likely to contain no large copy number aberrations. We identify the mean of all ratios for this set of intervals and translate the entire distribution of ratios so that this mean is equal to 1. The final step requires a predetermined amount of normal admixture, μ. Each ratio r is then scaled using the following linear transformation: $(r - 1)\frac{1}{1-\mu} + 1$.

P Breast Cancer Sequencing Data

The breast cancer samples analyzed were sequenced using Illumina paired-end technology with read length of 100bp or 108bp. We downloaded the BAM files from the European Genome-phenome Archive (`http://www.ebi.ac.uk/ega/`, accession number EGAD00001000138). The read depth vector is derived from the number of concordant paired reads where each read has a mapping quality ≥ 30.

We set the interval weight vectors $\mathbf{w}$ equal to to the read depth vector for the matched normal sample.

This is a reasonable weight vector use; for example, if $\frac{w_i}{w_j} = 2$ we expect the number of reads originating from a single copy of ith interval to be as twice as the number of reads originating from a single copy of jth interval. That is, $\frac{r_i}{r_j} \approx 2$, for a large enough number of reads and intervals i and j are not amplified or deleted in the tumor sample.

Q PD4120a - Further Analysis

In this section we present some additional results and further analysis of the sample PD4120a from [5] not included in the main text.

Q.1 $n = 2$ analysis

We present here the results of when we run our algorithm with $n = 2$ on sample PD4120a. We infer that the sample contains 34.3% normal cells and 65.7% tumor cells, compared to the 30% and 70% respectively reported by [5]. Fig S8 shows the complete set of copy number aberrations predicted using this analysis.

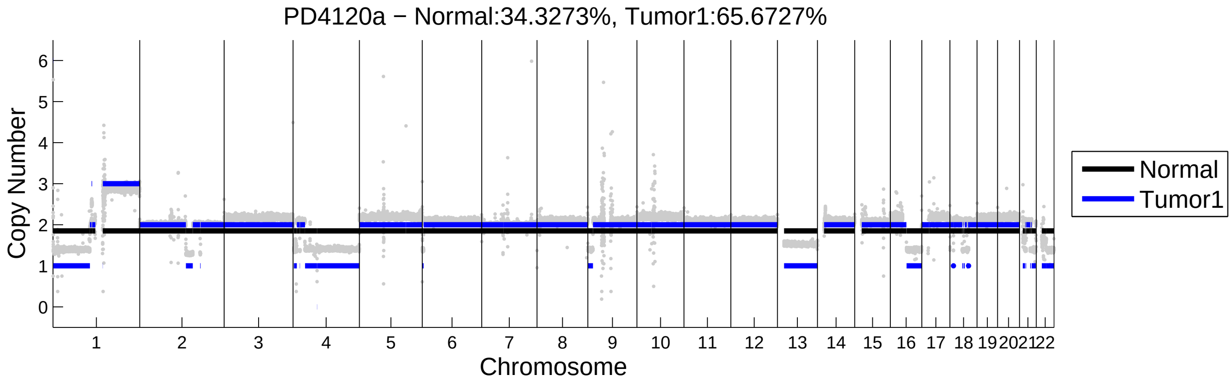

Figure S8: Sample PD4120a when we assume a single clonal population. We identify several abberations including the trisomy of 1q, and deletions of 1p, 4q, 13q, 16q and 22q. Copy numbers for the normal genome are in black and copy numbers for the tumor genome are in blue. Read depth ratios (gray) are over 50 Kb bins across the entire genome.

Q.2 $n = 3$ analysis

We performed analysis using our algorithm with $n = 3$ on two subsets of intervals in sample PD4120a. In the main text we presented results for the larger set of intervals which included the addition of all intervals for chromosome 22. Here we present the similar results obtained when using the original smaller set of intervals. Our algorithm estimates a normal admixture of 27.96% and two tumor populations comprising of 62.19% and 9.84% of the cells in the sample (Fig. S9). Again, we recover the fully clonal loss of 4q, and also estimate loss of 16q and part of 22q as clonal aberrations – the later [5] reports as subclonal. We also estimate the trisomy of 1q as subclonal - which [5] reports as clonal. We find a subclonal loss of 13q in 62.19% of the total cells in the sample. In comparison, [5] report a similar subclonal deletion in 47% of all cells in the sample (68% of the tumors cells in a 70% pure sample). Lastly, we identify subclonal deletions of chromosomes 8,11,12,14,and 15 in 9.84% of cells in the sample. [5] report aberrations for these chromosomes in 9.8% of the cells in the sample (14% of a 70% pure sample). However they claim that these

aberrations are chromosomal loss after a genome duplication, a scenario that is not found by our analysis.

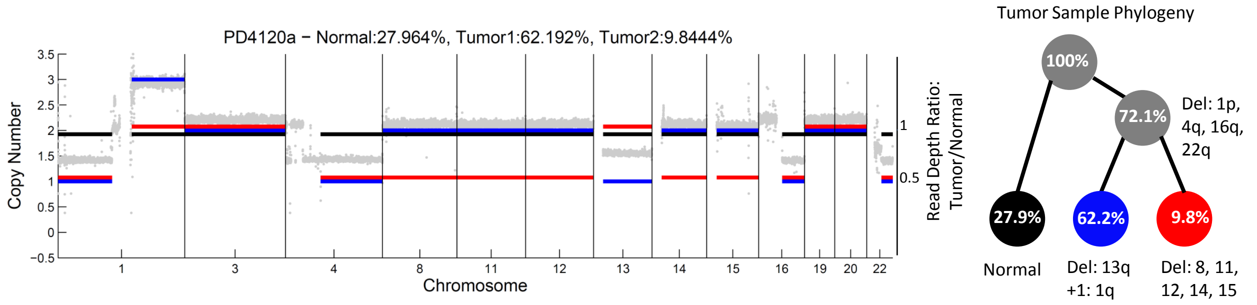

Figure S9: Analysis of copy number aberrations in 188X coverage breast tumor PD4120a with $n = 3$ for a smaller subset of intervals than presented in the main text. (Left) We show a copy number profile of of the normal population (black), dominant (clonal) tumor population (blue), subclonal tumor population (red) and read depth ratios (gray). (Right) A reconstruction of the tumor phylogeny with the estimated fraction of cells in each subpopulation. We identify copy number changes in chromosomes 1p, 4q, 16q, and 22q as clonal, the deletion of 13q and trisomy of 1q as part of the 62.2% subclonal population and deletions of chromosomes 8, 11,12,14,15 as part of the 9.8% subclonal population.

Additionally, we use estimates of normal admixture to correct read depth ratios over all chromosomes in order to predict subclonal events in chromosomes that were not used directly in the $n = 3$ analysis. We can predict the existence of a subclonal event if a peak in the read depth distribution occurs at a value that is not near a multiple of 0.5. Using this method we are able to predict the existence of subclonal deletions of chromosomes 2, 4p, 6, 7, 9, 18 and 21 (Fig. S10). The deletions in chromosomes 2 and 7 appear in a similar fraction of the sample, while the deletions in chromosomes 4p, 6, 7, 9, 18 and 21 appear in a similar fraction of the chromosomes – perhaps as part of the same subclonal population as the deletions of chromosomes 8,11,12,14, and 15 predicted directly by THetA.

Q.3 Comparisons between Our Predictions and [5]

In this section we provide additional details of differences between our predictions and those presented in [5]. One of the techniques we use to validate our predictions is to use corrected read depth ratios in 50 Kb intervals over entire genomes or specific genomic intervals. Figure S11(B) shows that such binning provides a more discerning view of read depth ratios than considering ratios over individual SNP positions (Figure S11(A)).

Chromosome 1q We performed further analysis of chromosome 1q for sample PD4120a. [5] indicated that the amplification of 1q to 3 copies is a clonal mutation (occurring in 70% cells), whereas our analysis finds that 1q is a subclonal mutation (occurring in 62% cells). We partitioned the genome into 50 Kb intervals and determined read depth vectors over all intervals for both the tumor sample and matched normal sample. We determined read depth ratios by normalizing the total number of reads to be the same for both the tumor and normal sample to be equal. Both our analysis and that done by [5] agree that chromosome 3 does not contain large copy number aberrations. We therefore center the ratios so that the average ratio in chromosome 3 is set to a ratio of 1 (Fig. S11(A)). We also look at how the distribution changes when we correct for 28% normal admixture (as estimated by our model) and 30% normal admixture (as predicted in

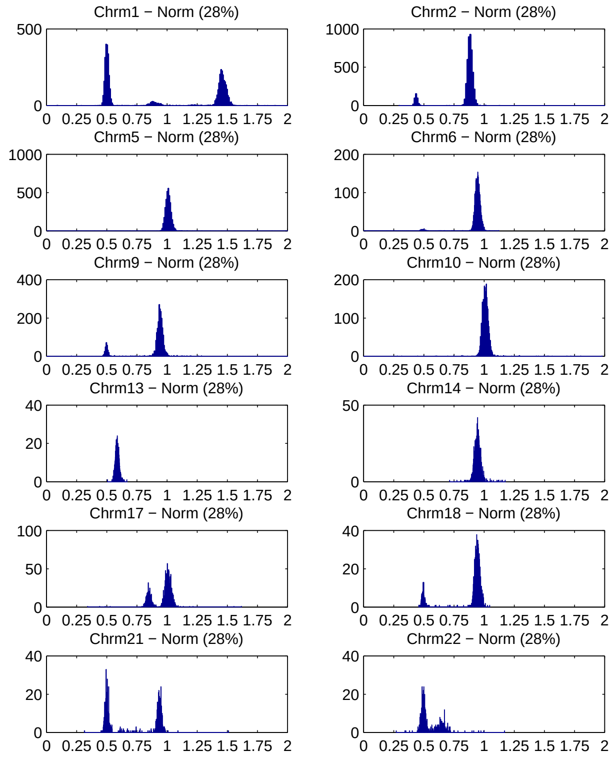

Figure S10: For each autosomal chromosome in PD4120a, the distribution of read depth ratios over 50 Kb intervals after centering and correction for 28% normal admixture for each using a simple linear scaling. Peaks that occur not at a ratio that is not divisible by 0.5 indicate the presence of a subclonal aberration.

[5]). In both of these cases, we see that the amplified intervals (which are those intervals in chromosome 1q) appear to be amplified in a smaller proportion of the cells than the clonal deletions (Fig. S11(B) and Main Text Fig. 3B). We analyze this data further by focusing on the intervals contained in the amplified portion of 1q and testing how far the corrected ratios are from the expected ratio of 1.5 (for an amplification to copy 3). We find that average ratio for intervals in 1q with normal admixture of 27.96% is 1.456 and with normal admixture of 30.0% is 1.47). While the later estimate is closer to the expected value of 1.5 (given copy number 3), the observed distributions have p-values of $4.7E - 80$ and $2.9E - 39$ using a a t-test when compared to the expected mean value of 1.5 – an indication that this aberration may indeed be subclonal.

We also perform further statistical analysis by comparing the read depth ratios for 1q to other intervals we predict to be clonally deleted (1p, 4q, and 16q). For all 4 intervals we use a linear scaling (see Main Text Methods) to correct read depth ratios for a range of possible *aberration fractions* – the fraction of the sample containing the aberration. For each aberration fraction and interval pair, we calculate a Z-score equal to $\frac{x-\mu}{\sigma}$ where x is the expected corrected ratio (0.5 for deletions, 1.5 for amplification of a single copy) for the interval and μ and σ are the mean and standard deviation of the corrected ratios. For all pairwise combinations such interval and aberrations fraction pairs, we calculate a pairwise Z-score by summing the absolute value of the corresponding Z-scores. Lower pairwise Z-scores indicate a better fit to the data. We investiage all pairwise Z-scores in Fig. S12 and find evidence that the aberrations in 1p, 4q and 16q are likely to occur in similar fractions of the sample. Whereas, the amplification in 1q appears to occur in a smaller fraction of the sample than the deletions in 1p, 4q and 16q. This evidence supports our hypothesis that the amplification of 1q was a subclonal event.

Chromosome 22 We performed further analysis of Chm22 in sample PD4120a which our analysis in the main paper indicates that 22q contains a clonal deletion as well as a subclonal deletion. [5] posit that a translocation between Chr1 and Chr22 is subclonally deleted. We used the algorithm GASV [7] to cluster discordant pairs where one read aligned to Chr1 and the other read aligned to Chr22. We find a cluster of 41 discordant supporting a single non-reciprocal translocation between Chr1p21 and Chr22q12. A visual representation [8] of these discordant read pairs is located in Fig S13(B). This finding supports another potential sequence of events, where the non-reciprocal translocation between Chr1 and Chr22 results in clonal deletion of parts of 1p and 22q and a dicentric chromosome. This is later followed by a subclonal deletion of a part of 22q which was not previously deleted in the translocation (Fig. S13). Dicentric chromosomes are known to be unstable, and so this deletion might occur on this chromosome.

Q.4 Analysis of PD4120a using ASCAT

We ran the ASCAT algorithm [9] on synthetic SNP array data we created from sequencing data for sample PD4120a [5]. For the 907,693 SNP locations on chromosomes 1 - 22 queried by the Affymetrix 6.0 SNP array, we created the two types of input used by ASCAT: 1) LogR values; and 2) B allele frequency (BAF). For each SNP position we counted the number of reads containing different alleles for both the tumor and matched normal samples. BAF values were calculated as the fraction of such reads containing the variant allele. LogR ratios for the were calculated by using quantile normalization [1] over the read counts for the matched tumor and normal sample and then taking the log_2 of the ratio of number of reads containing

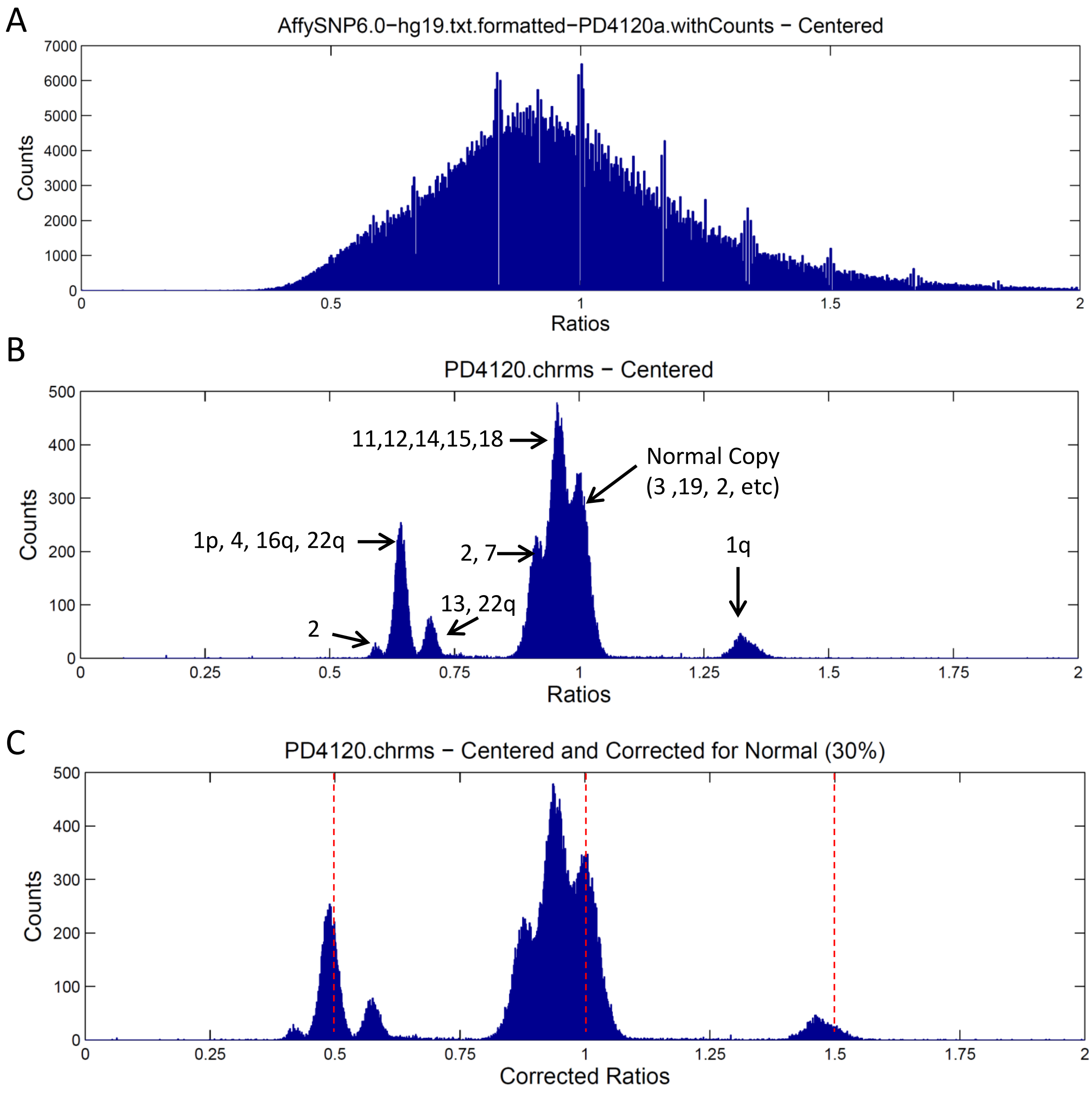

Figure S11: Distribution of read depth ratios for sample PD4120a. All plots are centered so that the mean ratio for Chromsome 3 (which doesn't contain larger copy number aberrations) is set to 1. **A.** Read depth ratios determined by counting the number of reads with an alignment including known germline SNP positions. **B.** Read depth ratios in 50 Kb bins. **C.** Read depth ratios after correction for 30% normal admixture using a simple linear scaling. The peak for chromosome 1q is less than 1.5, as would be expected if this aberration were clonal.

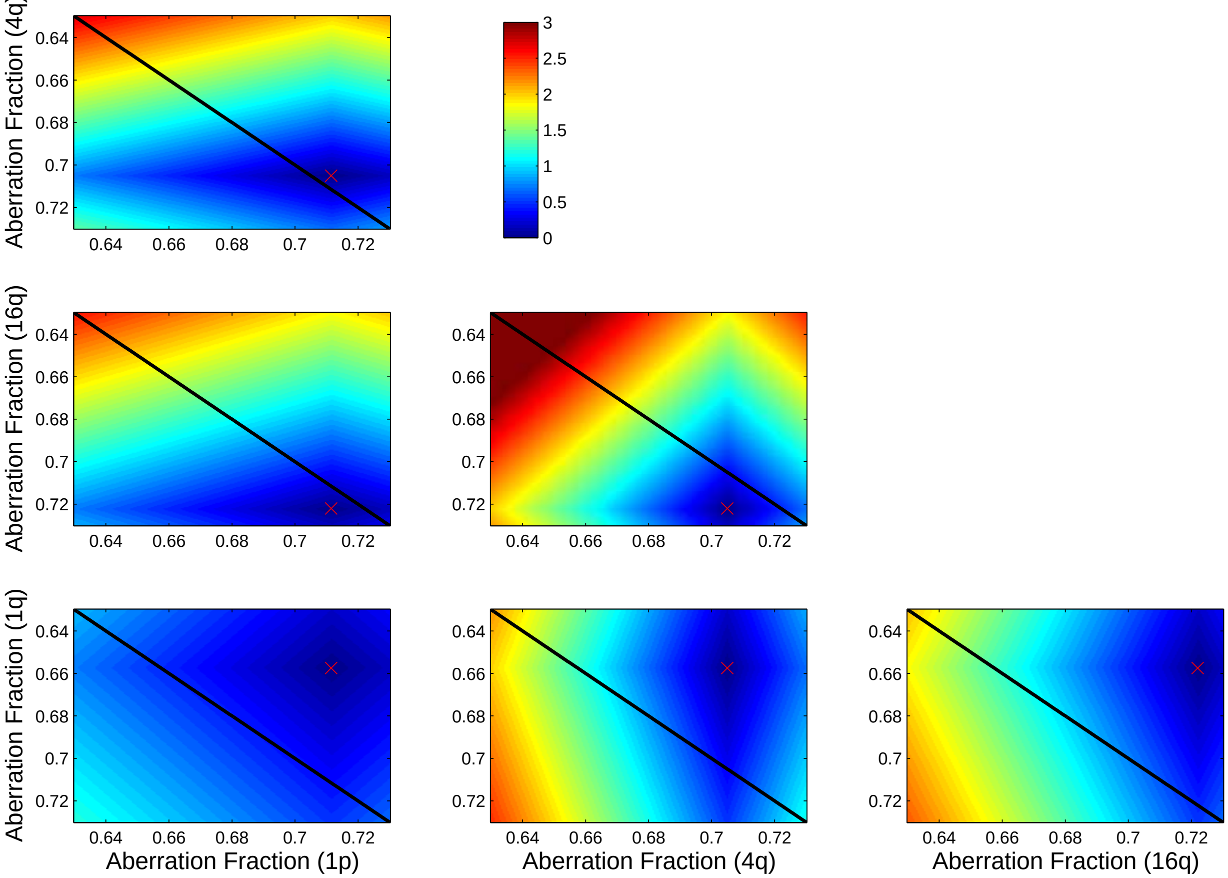

Figure S12: Pairwise Z-scores for corrected read depth ratios in aberrations occuring in sample PD4120a. A lower intensity indicates a lower Z-score, and an overall better fit with the data. The black diagonal marks equal aberration fractions between the intervals under consideration. The red X marks the pair of aberration fractions with the lowest pairwise Z-score. The closer that the red X is to the black diagonal, the more likely that the aberrations occur in a similar fraction of the sample and hence are in the same subclonal/clonal population.

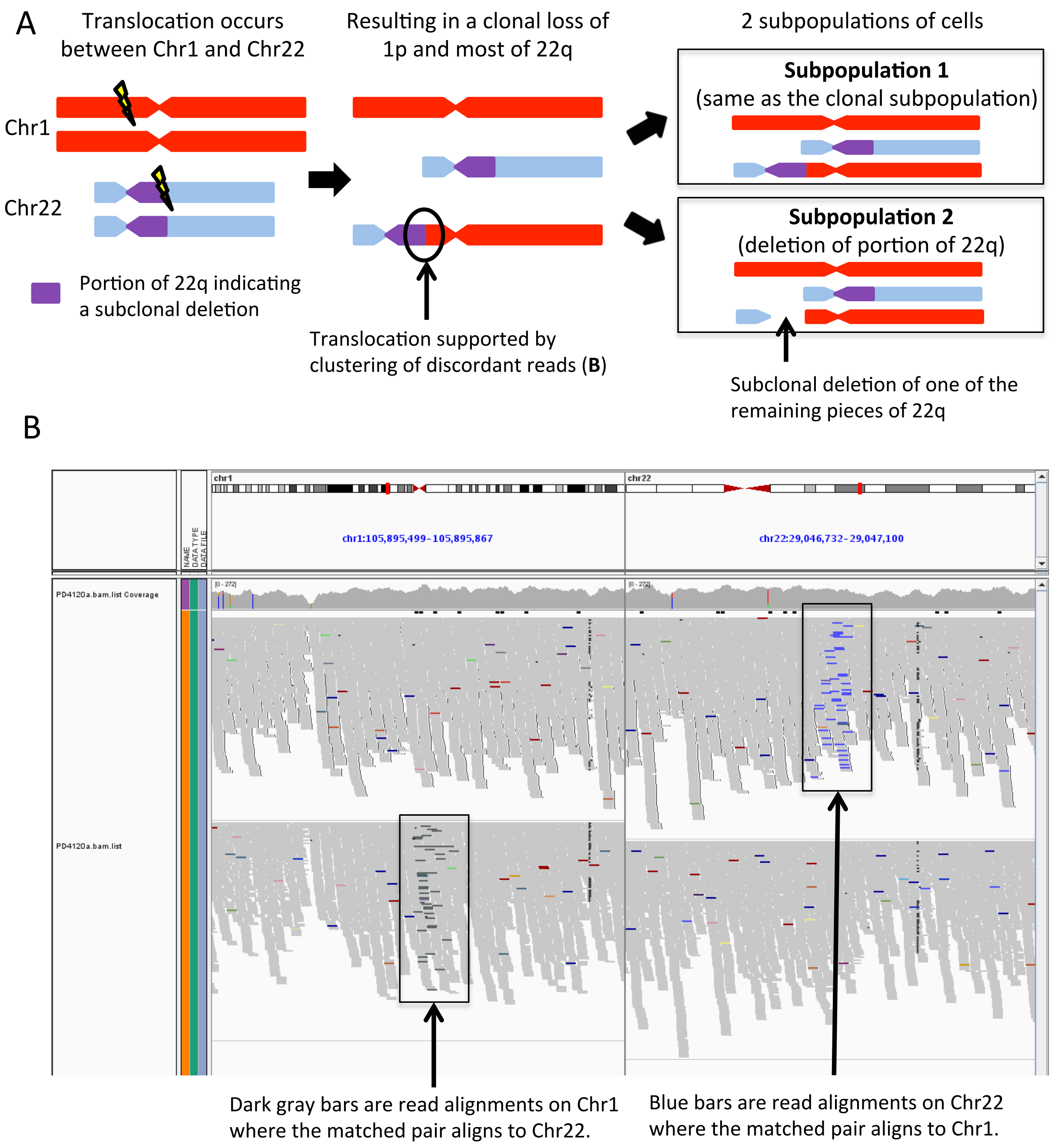

Figure S13: A. A potential sequence of events describing the clonal and subclonal deletions observed in 22q for sample PD4120a. A translocation (whose position is indicated by yellow markers) occurs between Chr1 (red) and Chr22 (blue) leading to a clonal loss of 1p and a portion of 22q. Then one of the two remaining copies of a portion of 22q (purple) is later subclonally deleted. **B.** Visual display of the discordant read pairs that support the translocation between Chr1 and Chr22.

the SNP location from the tumor sample over the number from the normal genome. By default, ASCAT assumes that LogR values have been scaled by a platform dependent parameter $\gamma = 0.55$, so we scaled all LogR values by this γ. LogR ratios for the normal sample were all set to 0. ASCAT inferred sample purity of 66% using this synthetic SNP data as input, a value similar to our estimate when $n = 2$ of 65.6%. The copy number aberrations predicted by ASCAT agree with both our analysis and that in [5] on several aberrations like the trisomy of 1q and the monosomy of 4q, but differs by predicting that chromosomes 17, 18, 19 and 20 are amplified.

R PD4115a - Further Analysis

In this section we present some additional results and further analysis of the sample PD4115a from [5] not included in the main text.

R.1 $n = 2$ analysis

We present here the results when we run our algorithm with $n = 2$ on sample PD4115a. On initial analysis we infer that the sample contains a high normal admixture and most copy numbers returned were equal to 1. Under our model, an equally likely solution is obtained by adding 1 to all copy numbers, thus translating the mode to copy number 2, and redetermining normal admixture. Using that sequence of steps, our algorithm reports that PD4115a contains 32.33% normal cells and 67.67% tumor cells (Fig. S14(A)). We identify several amplifications including 1q (+3), 7q (+1) and part of 8q (+5). We also identify multiple deletions including 9q, 11q, and 14q. Figure S14(B) shows the distribution of read depth ratios over the entire genome after centering using chromosome 20 (determined by the above analysis to have normal copy number of 2). Figure S14(C) shows the same distribution of read depth ratios but includes a correction for the estimate normal admixture of 32.33%. Other than the normal copy peak at a corrected ratio of 1, these corrected ratios do no align well to increments of 0.5, as would be expected if the sample was clonal. This supports our analysis that this sample contains several subclonal populations.

R.2 $n = 3$ analysis

We also ran our algorithm for $n = 3$ on a subset of the longest intervals in the BIC-Seq partition (since no chromosome was partitioned into a single interval). Due to the greater amount of fragmentation of this genome, we used a parameter of 0.4 for our heuristic for setting lower and upper bounds. Our analysis indicated that the sample contained two distinct subclonal populations in near similar proportions. We compare these subclonal populations using Z-scores in the same manner as described in the previous section. The only difference is that we compare a deleted interval in chromosome 3p simultaneously to the deleted intervals in 3q, 4q and 5q (Fig. S15). We find that the deletion in 3p does appear to be deleted in a different fraction of cells than the other deletions.

S PD4088a analysis

In this section we provide analysis of sample PD4088a, not contained in the main text. [5] report that sample PD4088a contains little subclonal copy number variation, (although they do not provide tumor purity estimates or copy number aberrations) making this sample a good candidate to analyze with our efficient method for inferring a clonal population. Following BIC-Seq segmentation ($\lambda = 200$), we find that chromosome 17

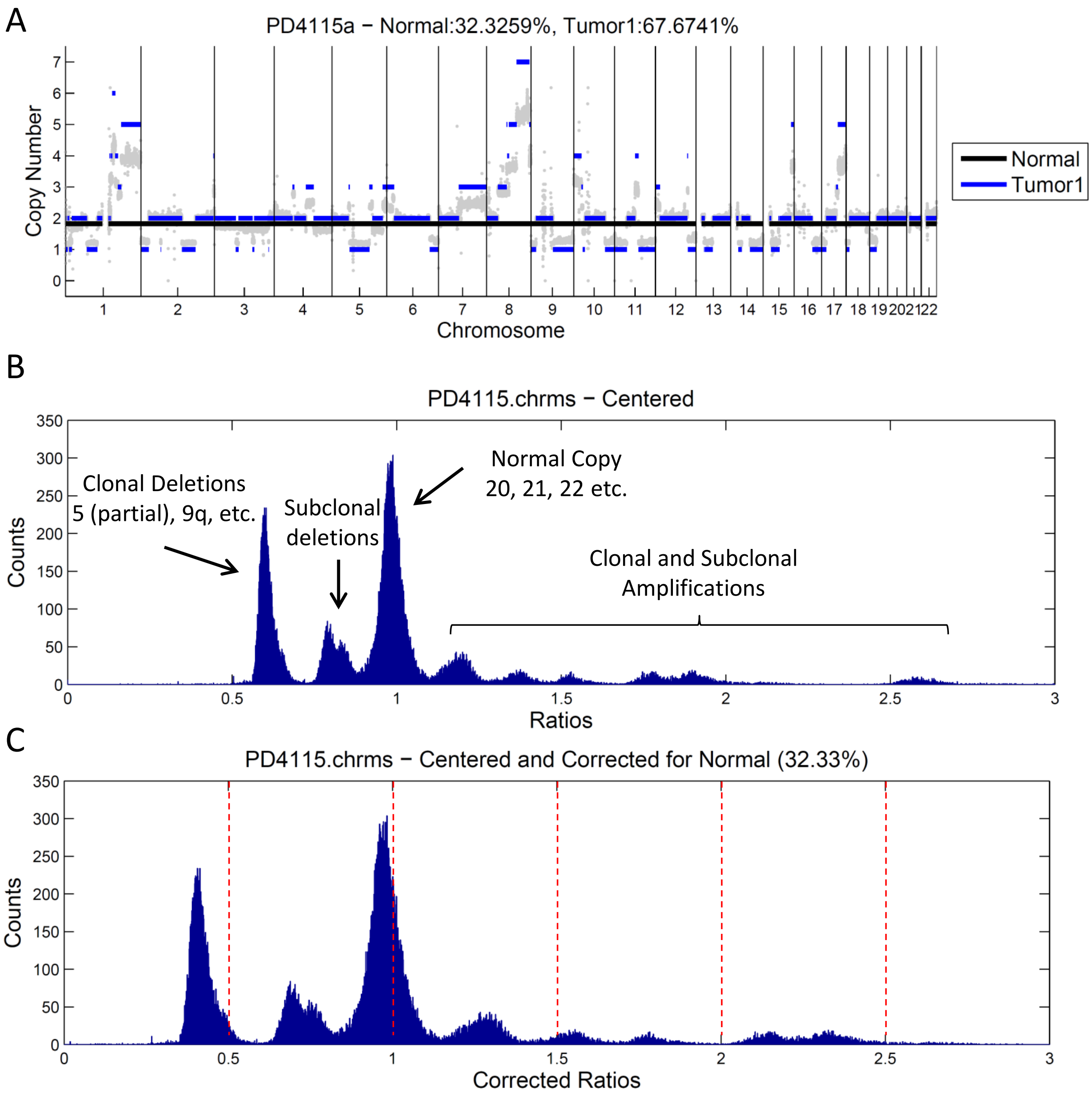

Figure S14: Distribution of read depth ratios when sample PD4115a is partitioned into 50 Kb intervals. (A) Read depth ratios when distribution is centered so that the mean ratio in Chromosome 20 is set to a ratio of 1. (B) Ratios after centering, and correction for 32.33% normal admixture using a simple linear scaling. No peaks fall near expected corrected ratios - supporting our analysis that this sample contains subclonal aberrations. (C) Inferred copy number aberrations by our algorithm when $n = 2$ including the normal population (black), clonal tumor population (blue), and read depth ratios (gray).

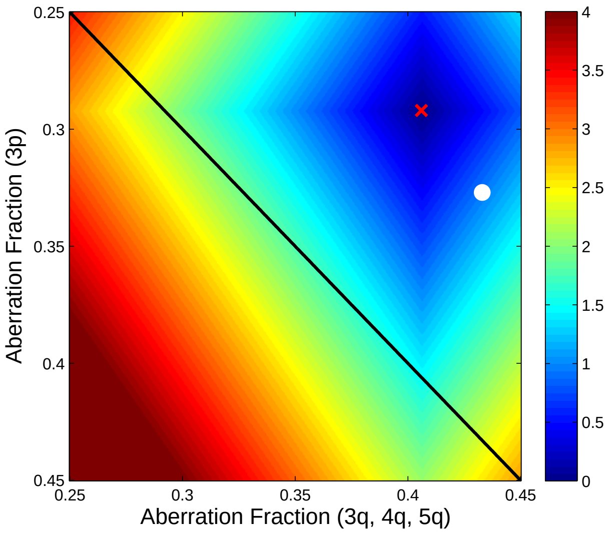

Figure S15: Pairwise Z-scores for corrected read depth ratios between different subclonal deletions PD4115a. A lower intensity indicates a lower Z-score, and an overall better fit with the data. The black diagonal marks equal aberration fractions between the intervals under consideration. The red X marks the pair of aberration fractions with the lowest pairwise Z-score. The closer that the red X is to the black diagonal, the more likely that the aberrations occur in a similar fraction of the sample and hence are in the same subclonal/clonal population. Here the red X is quite far from the black line - an indication that these deletions do occur in different subclonal populations. The white dot indicates the aberration fractions estimated by our algorithm and is near (within $\sim$1) the optimal pairwise Z-score within for this subset of deletions.

is extensively fragmented containing 25% of the intervals. To avoid overfitting to this chromosome, we remove all chromosome 17 intervals from consideration. Our algorithm run with $n = 2$ reports that PD4088a contains 41% normal cells and 59% tumor cells and identifies a loss of chromosomes 3, 10q, 11q, 18q, and 22q in the tumor population (Fig. S16(A)). Setting $n = 3$ and selecting a subset of intervals using the rules discussed in the main text (with an interval length lower bound of 40 Mb) also indicates that this sample is mostly clonal (Fig. S16(B)). This analysis indicates a normal admixture of 40.2% with two subclonal population comprising 58.6% and 1.2% of cells in the sample. Aberrations reported in the major subclonal population including a deletion of chromosomes 3, 10q, 11q, and 22q are the same as those reported in the clonal population in our analysis when $n = 2$. When we compare the $n = 2$ and $n = 3$ solutions using our version of the BIC model selection (see Methods) we choose the $n = 2$ solution indicating that this sample is mostly clonal.

Read depth ratio analysis also supports our conclusion that this sample is mainly clonal. Figure S16(C) shows the original distribution of tumor/normal read count ratios over 50KB bins across the genome. Figure S16(D) shows this distribution after correction for normal admixture using our estimates of the $n = 2$ analysis. The clearly defined peaks at corrected ratios of 0.5, 1 and to some extent at 1.5 indicated that this sample is indeed mostly clonal (at least with respect to copy number variants).

T Software

Our algorithm is implemented in Java and Matlab and is available at `http://compbio.cs.brown.edu/software/`. In our implementation we use the spectral projected gradient method provided by [6] to solve individual optimization problems.

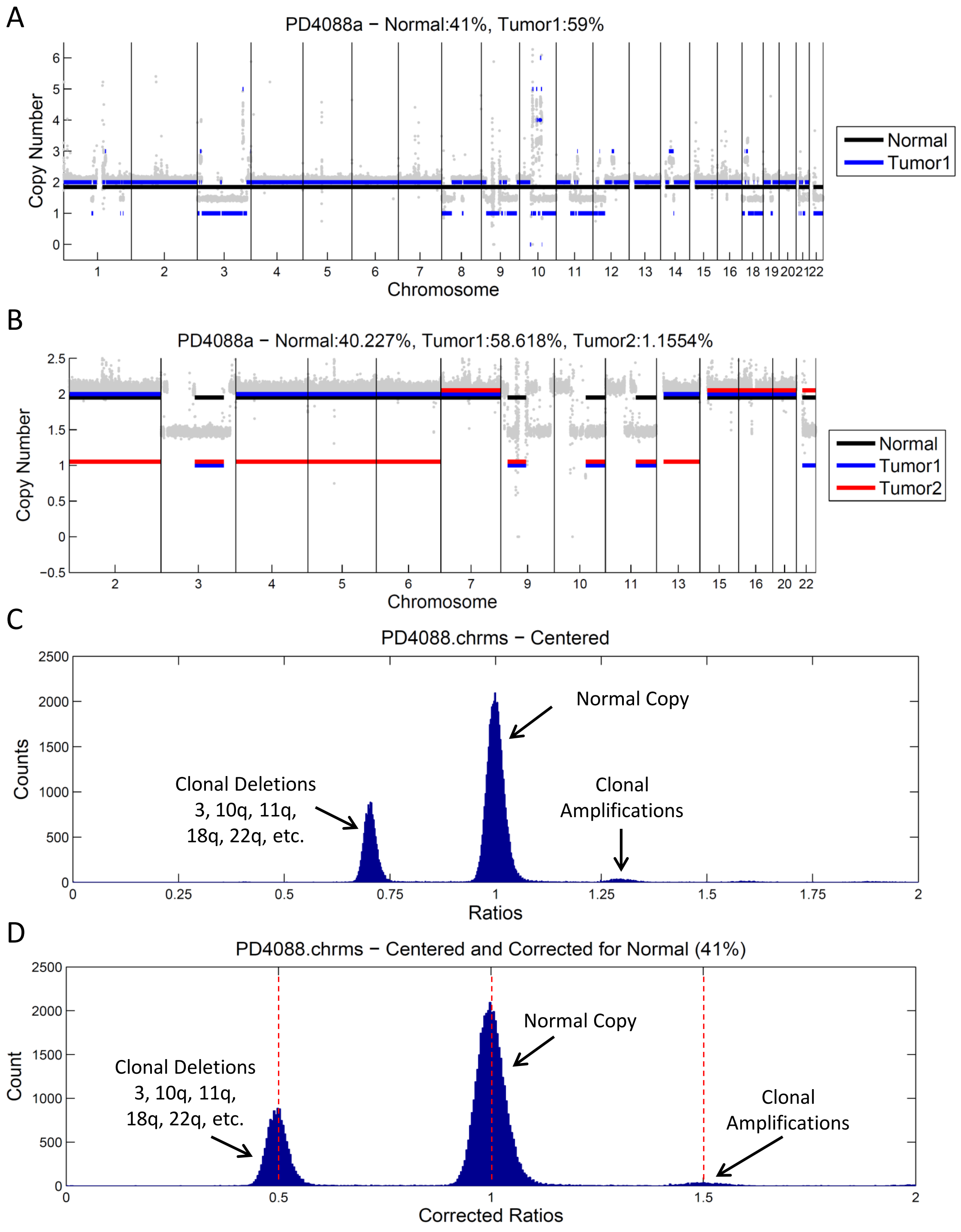

Figure S16: Analysis of the ∼40X coverage breast tumor PD4088a. **A.** Read depth ratios (gray) and the inferred copy number aberrations by our algorithm when $n = 2$ including the normal population (black), and clonal tumor population (blue). **B.** Read depth ratios (gray) and the inferred copy number aberrations by our algorithm when $n = 3$ including the normal population (black), major tumor population (blue) and subclonal population (red). **C.** Read depth ratios in 50KB intervals after centering so chromosomes 4,5,6 and 7 have a mean of 1. **D.** Read depth ratios in 50KB intervals after centering so chromosomes 4,5,6 and 7 have a mean of 1 and correcting for 41% normal admixture using a simple linear scaling. The only visible peaks fall near to expected corrected ratios (0.5, 1, 1.5), indicating that this sample is mostly clonal (with respect to copy number variants).

References

[1] B. M. Bolstad, R. A. Irizarry, M. Astrand, and T. P. Speed. A comparison of normalization methods for high density oligonucleotide array data based on variance and bias. *Bioinformatics*, 19(2):185–193, Jan 2003.

[2] S. L. Carter, K. Cibulskis, E. Helman, A. McKenna, H. Shen, T. Zack, P. W. Laird, R. C. Onofrio, W. Winckler, B. A. Weir, R. Beroukhim, D. Pellman, D. A. Levine, E. S. Lander, M. Meyerson, and G. Getz. Absolute quantification of somatic DNA alterations in human cancer. *Nat. Biotechnol.*, 30(5):413–421, May 2012.

[3] A. Gusnanto, H. M. Wood, Y. Pawitan, P. Rabbitts, and S. Berri. Correcting for cancer genome size and tumour cell content enables better estimation of copy number alterations from next-generation sequence data. *Bioinformatics*, 28(1):40–47, Jan 2012.

[4] T. J. Ley, C. Miller, L. Ding, B. J. Raphael, A. J. Mungall, A. Robertson, K. Hoadley, T. J. Triche, P. W. Laird, J. D. Baty, L. L. Fulton, R. Fulton, S. E. Heath, J. Kalicki-Veizer, C. Kandoth, J. M. Klco, D. C. Koboldt, K. L. Kanchi, S. Kulkarni, T. L. Lamprecht, D. E. Larson, L. Lin, C. Lu, M. D. McLellan, J. F. McMichael, J. Payton, H. Schmidt, D. H. Spencer, M. H. Tomasson, J. W. Wallis, L. D. Wartman, M. A. Watson, J. Welch, M. C. Wendl, A. Ally, M. Balasundaram, I. Birol, Y. Butterfield, R. Chiu, A. Chu, E. Chuah, H. J. Chun, R. Corbett, N. Dhalla, R. Guin, A. He, C. Hirst, M. Hirst, R. A. Holt, S. Jones, A. Karsan, D. Lee, H. I. Li, M. A. Marra, M. Mayo, R. A. Moore, K. Mungall, J. Parker, E. Pleasance, P. Plettner, J. Schein, D. Stoll, L. Swanson, A. Tam, N. Thiessen, R. Varhol, N. Wye, Y. Zhao, S. Gabriel, G. Getz, C. Sougnez, L. Zou, M. D. Leiserson, F. Vandin, H. T. Wu, F. Applebaum, S. B. Baylin, R. Akbani, B. M. Broom, K. Chen, T. C. Motter, K. Nguyen, J. N. Weinstein, N. Zhang, M. L. Ferguson, C. Adams, A. Black, J. Bowen, J. Gastier-Foster, T. Grossman, T. Lichtenberg, L. Wise, T. Davidsen, J. A. Demchok, K. R. Shaw, M. Sheth, H. J. Sofia, L. Yang, J. R. Downing, G. Eley, S. Alonso, B. Ayala, J. Baboud, M. Backus, S. P. Barletta, D. L. Berton, A. L. Chu, S. Girshik, M. A. Jensen, A. Kahn, P. Kothiyal, M. C. Nicholls, T. D. Pihl, D. A. Pot, R. Raman, R. N. Sanbhadti, E. E. Snyder, D. Srinivasan, J. Walton, Y. Wan, Z. Wang, J. P. Issa, M. Le Beau, M. Carroll, H. Kantarjian, S. Kornblau, M. S. Bootwalla, P. H. Lai, H. Shen, D. J. Van Den Berg, D. J. Weisenberger, D. C. Link, M. J. Walter, B. A. Ozenberger, E. R. Mardis, P. Westervelt, T. A. Graubert, J. F. DiPersio, and R. K. Wilson. Genomic and epigenomic landscapes of adult de novo acute myeloid leukemia. *N. Engl. J. Med.*, 368(22):2059–2074, May 2013.

[5] S. Nik-Zainal, P. Van Loo, D. C. Wedge, L. B. Alexandrov, C. D. Greenman, K. W. Lau, K. Raine, D. Jones, J. Marshall, M. Ramakrishna, A. Shlien, S. L. Cooke, J. Hinton, A. Menzies, L. A. Stebbings, C. Leroy, M. Jia, R. Rance, L. J. Mudie, S. J. Gamble, P. J. Stephens, S. McLaren, P. S. Tarpey, E. Papaemmanuil, H. R. Davies, I. Varela, D. J. McBride, G. R. Bignell, K. Leung, A. P. Butler, J. W. Teague, S. Martin, G. Jonsson, O. Mariani, S. Boyault, P. Miron, A. Fatima, A. Langerod, S. A. Aparicio, A. Tutt, A. M. Sieuwerts, A. Borg, G. Thomas, A. V. Salomon, A. L. Richardson, A. L. Borresen-Dale, P. A. Futreal, M. R. Stratton, and P. J. Campbell. The life history of 21 breast cancers. *Cell*, 149(5):994–1007, May 2012.

[6] M. Schmidt. minConf - a set of Matlab functions for optimization of differentiable real-valued functions subject to simple constraints on the parameters., 2008.

[7] S. Sindi, E. Helman, A. Bashir, and B. J. Raphael. A geometric approach for classification and comparison of structural variants. *Bioinformatics*, 25(12):i222–230, Jun 2009.

[8] H. Thorvaldsdottir, J. T. Robinson, and J. P. Mesirov. Integrative Genomics Viewer (IGV): high-performance genomics data visualization and exploration. *Brief. Bioinformatics*, 14(2):178–192, Mar 2013.

[9] P. Van Loo, S. H. Nordgard, O. C. Lingjorde, H. G. Russnes, I. H. Rye, W. Sun, V. J. Weigman, P. Marynen, A. Zetterberg, B. Naume, C. M. Perou, A. L. Borrensen-Dale, and V. N. Kristensen. Allele-specific copy number analysis of tumors. *Proc. Natl. Acad. Sci. U.S.A.*, 107(39):16910–16915, Sep 2010.

[10] R. Xi, A. G. Hadjipanayis, L. J. Luquette, T. M. Kim, E. Lee, J. Zhang, M. D. Johnson, D. M. Muzny, D. A. Wheeler, R. A. Gibbs, R. Kucherlapati, and P. J. Park. Copy number variation detection in whole-genome sequencing data using the Bayesian information criterion. *Proc. Natl. Acad. Sci. U.S.A.*, 108(46):E1128–1136, Nov 2011.
