# Supplementary figures and images for: THetA: inferring intra-tumor heterogeneity from high-throughput DNA sequencing data
Source: Genome Biol. 2013 Jul 29;14(7):R80. doi: 10.1186/gb-2013-14-7-r80 (PMC4054893; doi:10.1186/gb-2013-14-7-r80)

PD4120a – Normal:34.3%, Tumor1:65.7%

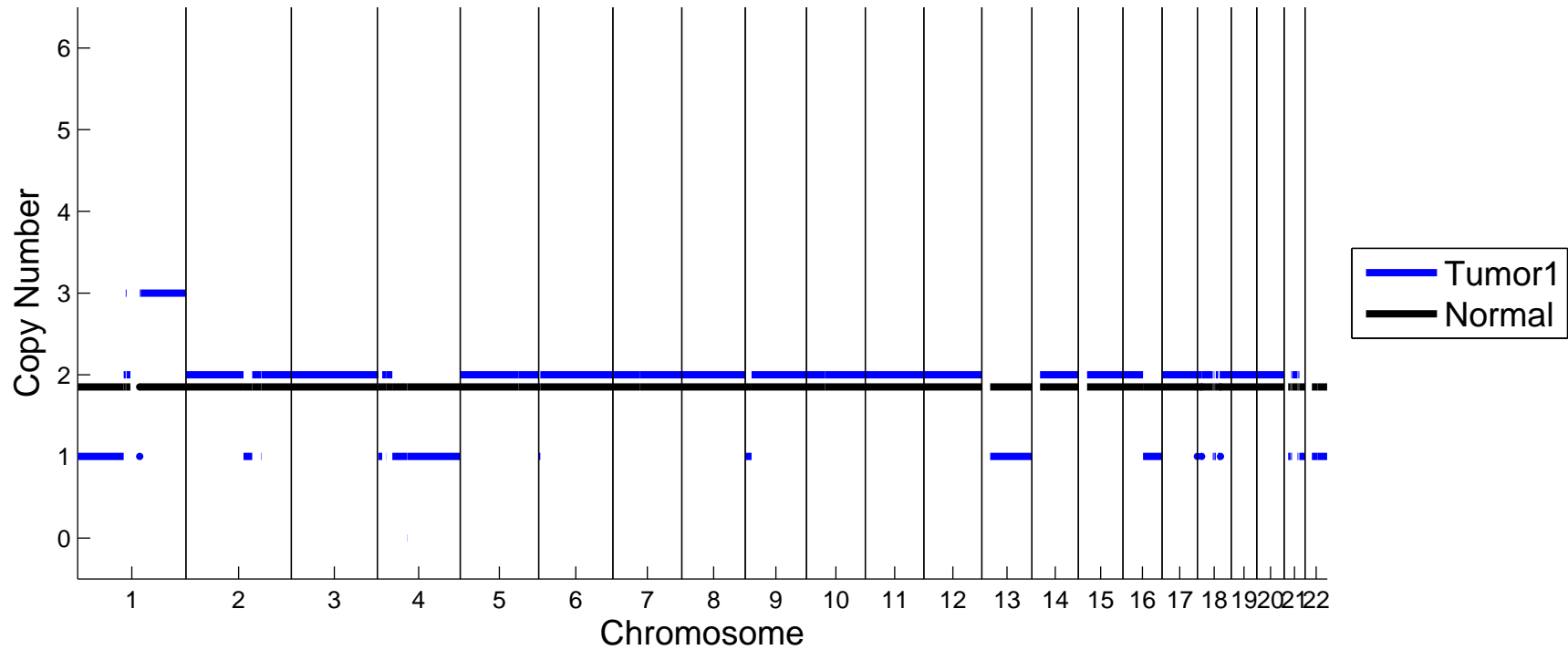

Supplement: Additional file 2 — THetA software package at the time of publication. In general, it is recommended that the latest version of THetA be downloaded from [60]. [file gb-2013-14-7-r80-S2.GZ › THetA_Beta_0.03_src/example/example.pdf]
